# Multi-omics machine learning identifies diagnostic gene signatures and functionally supports PRKACB involvement in macrophage inflammatory responses in sepsis

**DOI:** 10.3389/fimmu.2025.1611348

**Published:** 2026-01-15

**Authors:** Lvying Yang, Sisi Teng, Zengwen Ma, Cunqiao Han, Weiwei Qian

**Affiliations:** 1 Department of Respiratory and Critical Care Medicine, The First Veterans Hospital of Sichuan Province, Chengdu, Sichuan, China; 2 Department of Neurology, Shangjinnanfu Hospital, West China Hospital, Sichuan University, Chengdu, Sichuan, China; 3 Department of Emergency Medicine, Laboratory of Emergency Medicine, West China Hospital, and Disaster Medical Center, Sichuan University, Chengdu, Sichuan, China; 4 Department of Emergency, Shangjinnanfu Hospital, West China Hospital, Sichuan University, Chengdu, Sichuan, China

**Keywords:** biomarkers, machine learning algorithm, PRKACB, sepsis, single-cell RNA sequencing

## Abstract

Sepsis is a life-threatening condition caused by a dysregulated immune response, often leading to organ failure and death. Diagnosis and therapy remain challenging. This study aimed to identify biomarkers for sepsis through multi-omics analysis and experimental validation. A total of 1,166 samples from the GEO repository underwent differential analysis, WGCNA, and logistic regression to identify sepsis-associated features. After SVM-RFE screening, a 28-gene signature distinguishing sepsis from healthy controls achieved an AUC of 0.970 (sensitivity 0.939, specificity 1.000) in an independent cohort and 0.870 (sensitivity 0.906, specificity 0.700) in qRT-PCR validation. A 13-gene signature distinguishing sepsis from SIRS achieved an AUC of 1.000 (sensitivity 1.000, specificity 1.000) and 0.745 (sensitivity 0.500, specificity 0.900), respectively. PRKACB, consistently decreased in sepsis, emerged as a myeloid-associated hub gene with functional support in macrophages. Single-cell analysis revealed that PRKACB is predominantly expressed in myeloid cells, with macrophages and neutrophils showing the most notable compositional changes. Pathway enrichment indicated distinct PRKACB-related functions between sepsis and healthy controls, and across myeloid subtypes. Reducing PRKACB expression significantly upregulated TNF-α and IL-1β release and decreased THP-1 macrophage viability, suggesting that excessive inflammation may trigger apoptosis or metabolic dysfunction. These data support PRKACB as a myeloid hub linked to macrophage inflammatory output and viability, while defining the precise downstream signaling as a priority for future studies.

## Introduction

Sepsis is a life-threatening syndrome caused by an excessive and dysregulated immune response to infection, leading to systemic inflammation, organ failure, and high mortality (1, 2). Despite therapeutic advances, sepsis still affects over 30 million people annually, with mortality exceeding 25-50% (3, 4). Recent multi-omics and single-cell studies have revealed profound immunometabolic reprogramming and immune cell exhaustion in sepsis, uncovering potential diagnostic and therapeutic targets (5, 6). However, current treatments remain largely supportive, mainly antibiotics, fluid resuscitation, and oxygen therapy, because no targeted drugs are available (7, 8). Therefore, identifying reliable biomarkers and actionable therapeutic targets is crucial to improve early detection and precision treatment of sepsis.

Although sepsis is conceptually distinguished from sterile systemic inflammation, its clinical presentation substantially overlaps with systemic inflammatory response syndrome (SIRS) and other critical/inflammatory conditions, making early discrimination challenging (4, 9). Sepsis has historically been framed within the context of systemic inflammation accompanied by evidence of infection, as exemplified by prior clinical definitions that pair SIRS features with microbiological confirmation (10), and it is frequently accompanied by organ dysfunction (11, 12). However, commonly used biomarkers and reported transcriptomic candidates, including C-reactive protein, procalcitonin, and genes such as CD177, CYSTM1, and MMP8, often show limited robustness or inconsistent validation for reliably separating sepsis from clinically confusable states in real-world settings (13, 14). Therefore, there remains a clear unmet need for host-response molecular signatures that can accurately distinguish sepsis from SIRS and other overlapping inflammatory illnesses, thereby improving diagnostic precision and enabling more actionable stratification.

**Figure 1:**
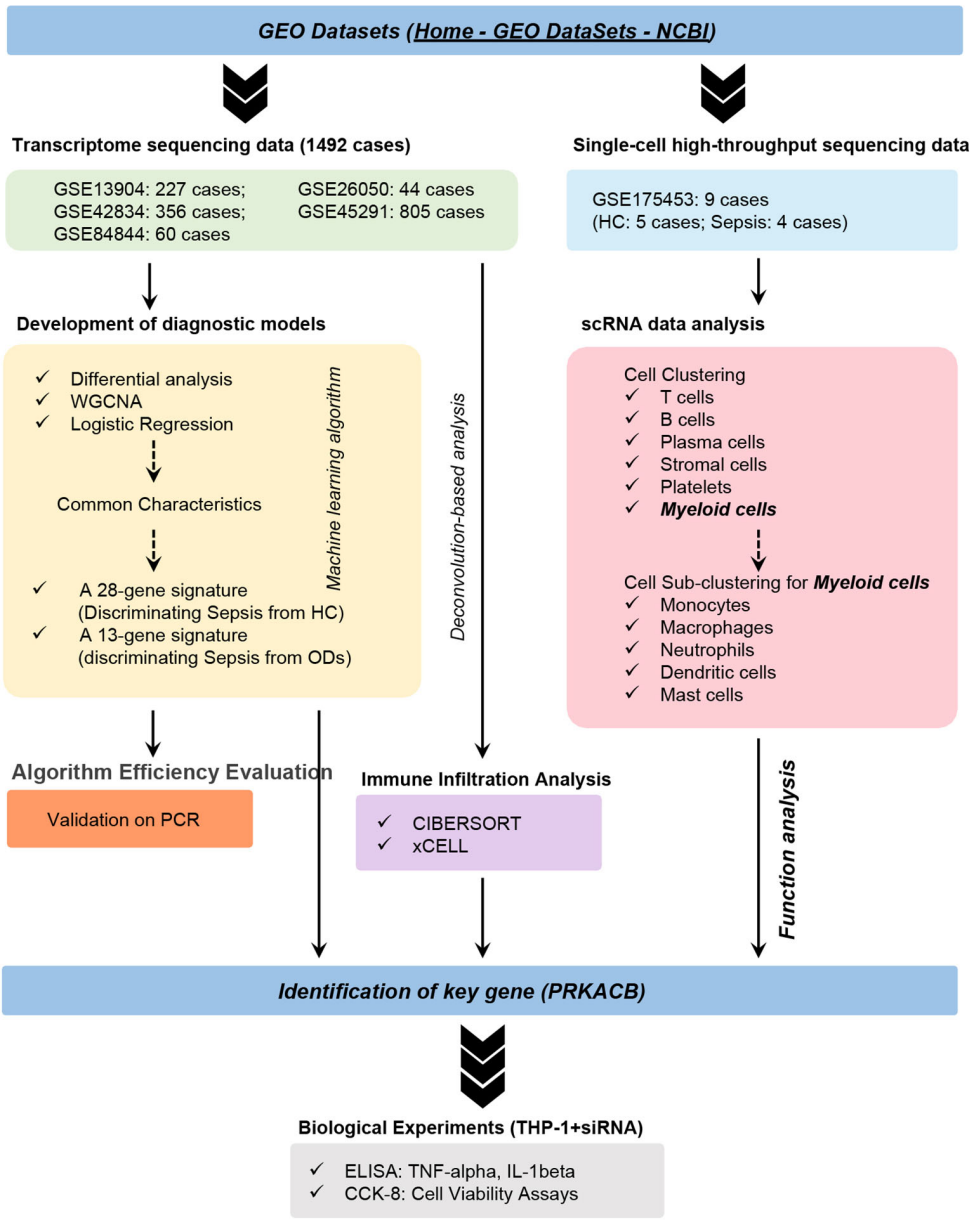
Workflow of the study. HC, healthy control; ODs, other diseases; WGCNA, weighted gene co-expression network analysis; GEO, Gene Expression Omnibus; scRNA-seq, single-cell RNA sequencing.

This work focuses on diagnostic and differential-diagnostic modeling rather than prognostic risk stratification. In recent years, machine learning has emerged as a powerful tool in biomedical research, enabling high-dimensional data integration and improving diagnostic accuracy across complex diseases (15, 16). Here, we applied SVM-RFE combined with logistic regression to develop two host-response signatures for sepsis. A 28-gene panel was built for primary diagnosis against healthy controls, and a 13-gene panel was constructed for differential diagnosis against clinically similar conditions, with external validation including qRT-PCR. Building on signature components, single-cell localization, and network analyses, we prioritized PRKACB as a myeloid-associated hub gene and performed macrophage knockdown experiments to provide functional support linking PRKACB to inflammatory outputs and cell viability. The workflow is shown in **Figure 1**.

## Materials and methods

### Data collection and preprocessing

A total of eight transcriptome sequencing datasets (GSE13904, GSE26050, GSE42834, GSE45291, GSE84844, GSE26378, GSE72326, and GSE66099), comprising 1,166 samples, were retrieved from the Gene Expression Omnibus (GEO) repository (https://www.ncbi.nlm.nih.gov/geo/). Raw expression matrices were log2-transformed and normalized using the *limma* package, and batch effects among datasets were corrected with the *ComBat* function in the *sva* R package. Detailed information about the datasets is provided in **Table 1**. Additionally, scRNA-sequencing dataset, GSE175453, was used to analyze immune cell distribution, target gene expression patterns, and explore the potential functions of the target gene. Differential expression analysis was conducted to identify genes significantly associated with sepsis or SIRS. Differentially expressed genes (DEGs) were determined with an adjusted *P* value < 0.05 and |log2(fold change)| > 1 as the cut-off criteria.

**Table 1:** Clinical characteristics of different cohorts.

Term	GSE13904	GSE26050	GSE42834	GSE45291	GSE84844	GSE26378	GSE72326	GSE66099
Basic Information								
Dataset	Array	Array	Array	Array	Array	Array	Array	Array
Platform	Affymetrix Human Genome U133 Plus 2.0 Array	Affymetrix Human Genome U133 Plus 2.0 Array	Illumina HumanHT-12 V4.0 expression beadchip	Affymetrix HT HG-U133+ PM Array Plate	Affymetrix Human Genome U133 Plus 2.0 Array	Affymetrix Human Genome U133 Plus 2.0 Array	Illumina HumanHT-12 V4.0 expression beadchip	Affymetrix Human Genome U133 Plus 2.0 Array
Tissue	Whole blood	PBMC	Whole blood	Whole blood	Whole blood	Whole blood	Whole blood	Whole blood
Data Structure/Cases								
HC	18	33	113	20	30	21	20	47
SIRS	22	na	na	na	na	na	na	30
Sepsis	99	na	na	na	na	82	na	199
HLH	na	11	na	na	na	na	na	na
Lung Cancer	na	na	16	na	na	na	na	na
Pneumonia	na	na	14	na	na	na	na	na
SA	na	na	39	na	na	na	na	na
TB	na	na	35	na	na	na	na	na
SLE	na	na	na	70	na	na	157	na
RA	na	na	na	60	na	na	na	na
pSS	na	na	na	na	30	na	na	na

PBMC, Peripheral Blood Mononuclear Cells; HC, healthy controls; SLE, Systemic Lupus Erythematosus; SIRS, Systemic Inflammatory Response Syndrome; SA, Sarcoidosis; RA, Rheumatoid Arthritis; pSS, Sjogren’s Syndrome; HLH, Hemophagocytic Lymphohistiocytosis; TB, tuberculosis.

### Weighted gene co-expression network analysis

Weighted Gene Co-expression Network Analysis (WGCNA) was performed to identify co-expression modules associated with sample traits. To construct the gene co-expression network, we referenced the article by Li et al. (17). A soft-thresholding power (β) of 5 was chosen based on the criterion of scale-free topology, as indicated by the scale-free topology model fit.

### Univariate logistic regression analysis

Univariate logistic regression analysis was conducted to evaluate the association between individual variables and sepsis. Each variable was analyzed separately, and odds ratios (ORs) along with corresponding 95% confidence intervals (CIs) were calculated. A p-value of less than 0.05 was considered statistically significant for identifying variables associated with the outcome. Variables meeting the criteria for differential expression analysis and closely related to sepsis were selected for the development of diagnostic signatures.

### Construction of diagnostic signature

Support Vector Machine-Recursive Feature Elimination (SVM-RFE) was used for feature selection in the training cohort, and two logistic regression diagnostic models were constructed to distinguish sepsis from healthy controls and from other diseases, respectively. Detailed procedures, including cross-validation, permutation testing, and batch correction settings, are described in the **Supplementary Text**.

### Identification of hub genes

Difference analysis was performed between SIRS and HC, as well as between sepsis and SIRS, to identify DEGs. These DEGs were analyzed using the STRING platform (https://cn.string-db.org) to construct a protein-protein interaction (PPI) network with a connectivity degree greater than 0.8. Additionally, we utilized xCell, and CIBERSORT platform to explore the immune functions associated with the core genes. We also established a ceRNA (mRNA-miRNA-lncRNA) network centered on the core genes using Cytoscape software (version 3.9.1), incorporating information from miRDB (https://mirdb.org), miRanda (http://www.microrna.org), and SpongScan (http://spongescan.rc.ufl.edu/).

### Single-cell RNA sequencing analysis

The GSE175453 single-cell transcriptome dataset was processed with Cell Ranger (v6.1.2) and analyzed in Seurat (v5) with quality control and doublet removal, SCTransform normalization, Harmony integration, clustering, and MAST differential expression, with full parameters provided in the **Supplementary Text**.

### Sample collection

A total of 70 whole blood samples were collected from Sichuan Provincial People’s Hospital, including 30 healthy individuals, 20 SIRS patients, and 20 sepsis patients. All blood samples were drawn using sterile techniques and stored in EDTA tubes for further analysis. SIRS, not caused by bacterial infection, was diagnosed using published criteria (9, 18). Sepsis patients were identified based on SIRS criteria, combined with a confirmed or suspected infection and an acute increase of ≥2 points in the Sequential Organ Failure Assessment (SOFA) score, indicating organ dysfunction. Informed consent was obtained from all participants prior to sample collection, and the study protocol received approval from the hospital’s ethics committee.

### Quantitative real-time PCR

All human blood samples used for qRT-PCR validation were collected with informed consent and approved by the Ethics Committee of the Second Affiliated Hospital of Hainan Medical University (approval No. Lunshen [Yan] 2024-536). Whole blood samples collected from HC, SIRS and sepsis patients were processed for RNA extraction using the TRIzol LS Reagent (Invitrogen, USA) following the manufacturer’s protocol. Total RNA was reverse-transcribed into cDNA using the PrimeScript™ RT reagent kit (Takara, Japan) according to the manufacturer’s protocol. Quantitative real-time PCR (qRT-PCR) was performed on the LightCycler^®^ 480 System (Roche, Switzerland) using the SYBR^®^ Premix Ex Taq™ II (Takara, Japan). The expression levels of target genes were quantified using gene-specific primers. The primer sequences used in the work were presented in **Supplementary Table 1**.

### Western blotting

Total proteins were extracted from cells using RIPA buffer supplemented with protease inhibitors, quantified by BCA assay, and separated by 10% SDS-PAGE. After electrophoresis, proteins were transferred to PVDF membranes, blocked with 5% non-fat milk, and incubated overnight at 4°C with a primary antibody against PRKACB (FNab06778, Wuhan Fine Biotech Co., 1:1000 dilution). Membranes were then probed with HRP-conjugated secondary antibody (1:5000) for 1 h at room temperature, and signals were detected using ECL substrate. GAPDH served as the loading control.

### Enzyme-linked immunosorbent assay

Cytokine levels were measured using commercial ELISA kits (Merck, Germany) according to the manufacturer’s instructions. Briefly, cell culture supernatants or tissue homogenates were centrifuged (10,000 × g, 10 min, 4°C) to remove debris. Standards and samples were added to pre-coated 96-well plates and incubated for 2 h at room temperature. After washing with PBS-Tween, biotinylated detection antibodies were added (1 h incubation), followed by streptavidin-HRP conjugate (30 min). Color development was initiated by TMB substrate, stopped with 1 M H_2_SO_4_, and absorbance was measured at 450 nm (reference: 570 nm). Cytokine concentrations were calculated from standard curves.

### Transfection

THP-1 cells were cultured in RPMI-1640 with 10% FBS and 1% penicillin/streptomycin. For differentiation, cells (5×10^5^ cells/mL) were treated with 100 nM PMA for 24 hours, rested for 24 hours, then activated with 100 ng/mL LPS (24 hours or 48 hours). Knockdown was performed using two siRNA duplexes targeting *PRKACB*: siPRKACB-1: 5′-GCAUUGAGACUGUGGUCUAtt-3′; siPRKACB-2: 5′-CCAGAGAUUCUACCGACAAtt-3′. Transfection was conducted with Lipofectamine 3000 (Invitrogen): siRNA (20 nM)/reagent complexes (Opti-MEM, 15 min incubation) were added to differentiated THP-1 cells for 6 hours before medium replacement. Knockdown efficiency was confirmed at 48 hours by qPCR and Western blot. Scrambled siRNA served as negative control.

### Statistical analysis

Differential expression analysis was performed using R software (version 4.4.1). The “limma” package was employed to identify differentially expressed genes (DEGs) between groups, with thresholds set at |log_2_(foldchange)| > 1 and adjusted *p*-value < 0.05, unless otherwise specified. For qRT-PCR data, statistical analysis was performed using GraphPad Prism (version 6.01). Relative gene expression levels were calculated using the 2^-ΔΔCt method, and the data were normalized to GAPDH expression. The results were analyzed using Student’s t-test for two-group comparisons. All *p*-values < 0.05 were considered statistically significant. Data are presented as the mean ± standard deviation (SD) from at least three independent experiments.

## Results

### Identification of differentially expressed genes

**Figure 2:**
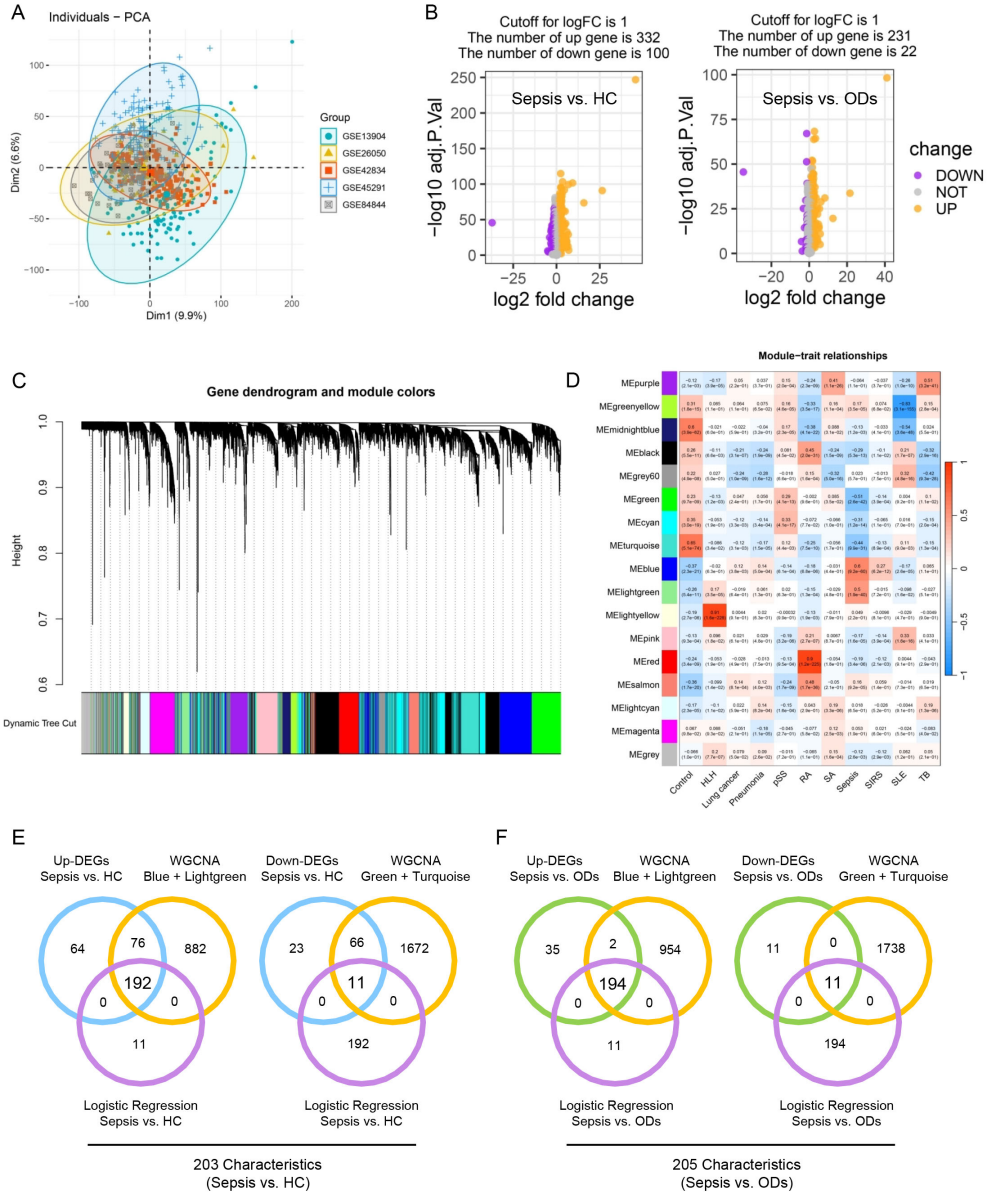
Identification of differentially expressed genes. **(A)** Samples distribution in a combined cohort (GSE13904, GSE26050, GSE42834, GSE45291, and GSE84844). **(B)** Difference analysis comparing sepsis with HC (left) and sepsis with ODs (right). **(C)** WGCNA, where genes are grouped into distinct modules through hierarchical clustering, with each color representing a different module. **(D)** The correlation of WGCNA modules with patient characteristics. **(E, F)** Identification of DEGs selected through univariate logistic regression analysis and present in WGCNA modules closely related to sepsis. SLE, Systemic Lupus Erythematosus; SIRS, Systemic Inflammatory Response Syndrome; SA, Sarcoidosis; RA, Rheumatoid Arthritis; pSS, Sjogren’s Syndrome; HLH, Hemophagocytic Lymphohistiocytosis; TB, tuberculosis; DEGs, differentially expressed genes.

After data integration and cleaning, we obtained a total of 610 samples, which included 214 normal samples, 99 sepsis samples, and 297 OD samples (**Figure 2A**). Based on difference analysis, 432 DEGs were identified in sepsis patients compared to HC, including 332 up-regulated and 100 down-regulated genes (**Figure 2B**). Additionally, there were 253 DEGs (231 up-regulated and 22 down-regulated) between sepsis patients and SIRS individuals. WGCNA was performed on the 610 samples to discover distinct modules associated with sepsis through hierarchical clustering (**Figure 2C**). WGCNA adhered to a scale-free topology model, with β = 5 chosen as the soft threshold based on average connectivity and scale independence (**Supplementary Figures 1A, B**). The analysis revealed that genes in the blue and light green modules were most positively associated with sepsis, while genes in the green and turquoise modules were negatively correlated with sepsis (**Figure 2D**).

To comprehensively identify genes significantly associated with sepsis, univariate logistic regression analysis was conducted among HC, sepsis patients, and OD individuals. A total of 203 characteristics were identified to distinctly discriminate sepsis from HC (**Supplementary Table 2**), and 205 characteristics were found to significantly differentiate sepsis from OD samples (**Supplementary Table 3**). Notably, the genes selected through univariate logistic regression analysis were all differentially expressed and were present in WGCNA modules closely related to sepsis (**Figures 2E, F**).

### Development and validation of diagnostic models for sepsis

**Figure 3:**
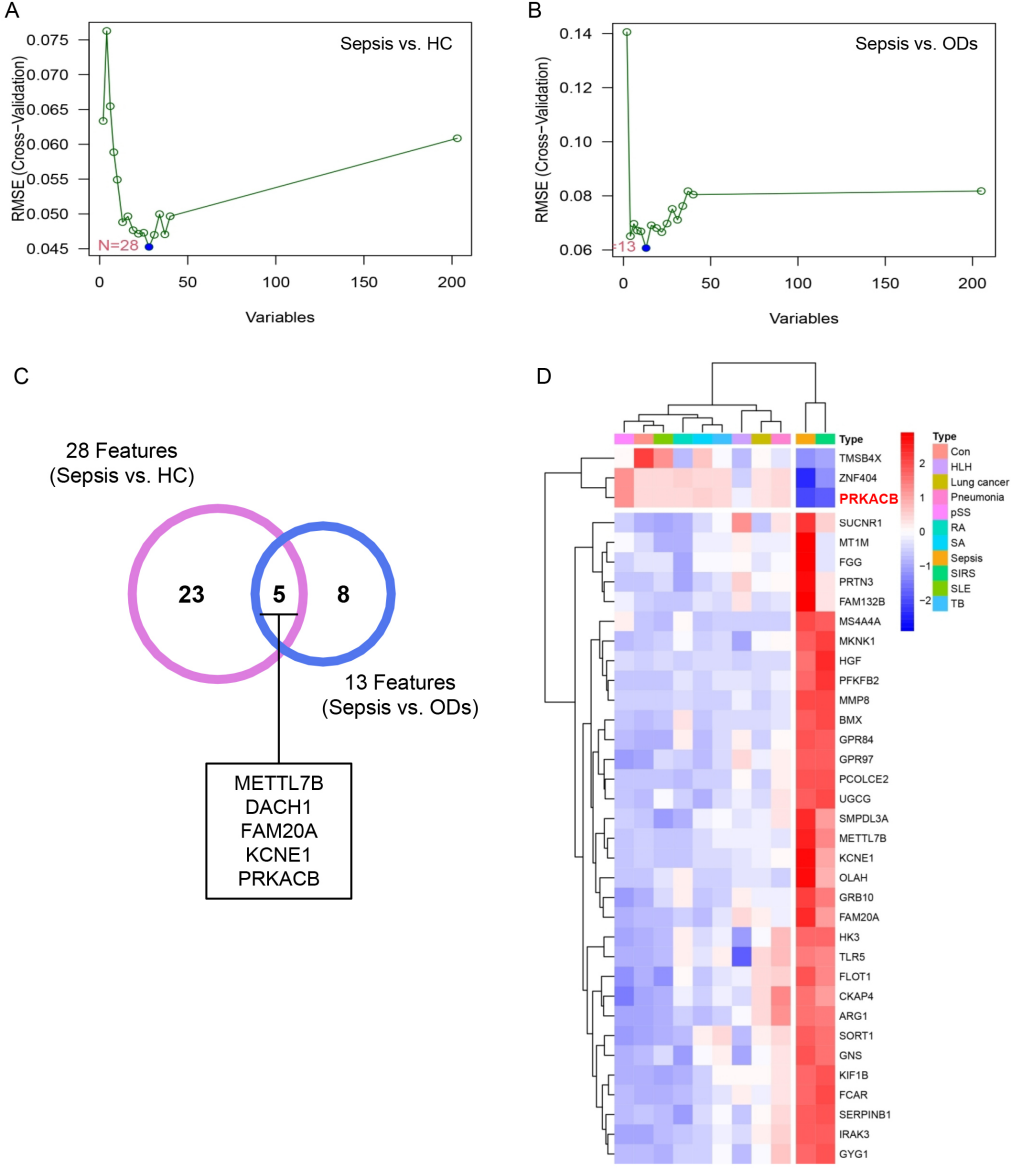
Identification of key features for discriminating sepsis patients. **(A)** Identification of 28 features differentiating sepsis patients from HC via SVM-RFE algorithm. **(B)** Identification of 13 features distinguishing sepsis from ODs via SVM-RFE. **(C)** Five common features. **(D)** Expression profiles of the features across various diseases. SVM-RFE, Support Vector Machine-Recursive Feature Elimination.

A total of 28 features were identified for distinguishing sepsis from HC based on SVM-RFE algorithm, while 13 features were identified for differentiating sepsis from ODs using the same method (**Figures 3A, B**). Notably, METTL7B, DACH1, FAM20A, KCNE1 and PRKACB were the common factors shared by both feature sets (**Figure 3C**), and the expression profiles of these features across different diseases are shown in **Figure 3D**.

**Figure 4:**
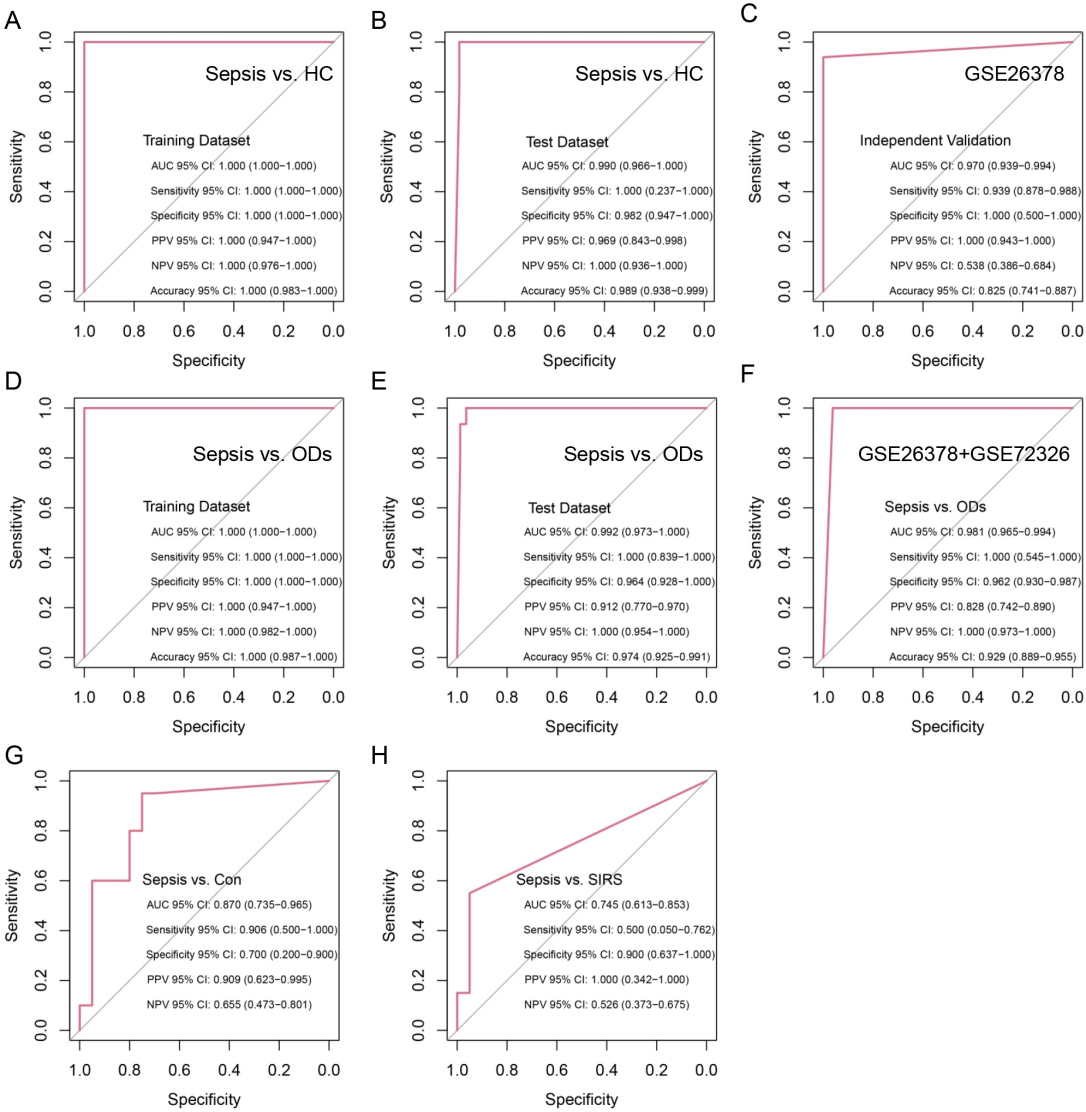
Identification and validation of diagnostic models for sepsis. **(A, B)** Development of a 28-gene signature distinguishing sepsis from HC in the training **(A)** and test **(B)** cohorts. **(C)** Independent validation of the 28-gene signature in GSE26378 cohort. **(D, E)** Development of a 13-gene signature discriminating sepsis from ODs in the training **(D)** and test **(E)** datasets. **(F)** Independent validation of the 13-gene signature in a combined cohort. **(G, H)** Experimental validation by qRT-PCR showing the signatures’ performance in differentiating sepsis from HC (28-gene panel) **(G)** and SIRS (13-gene panel) **(H)**. ROC curves were generated from the logistic-regression risk scores. AUC values with 95% confidence intervals were calculated using the pROC package (DeLong method). Sensitivity and specificity were computed using a single cutoff determined in the training cohort by Youden’s index and then applied unchanged to the test and validation cohorts.

The 28 features were then used to construct a logistic regression model (28-gene signature), achieving an AUC, sensitivity, and specificity of 1.000 in the training cohort for discriminating sepsis from HC (**Figure 4A**). In test cohort, the model yielded an AUC of 0.990, sensitivity of 1.000, and specificity of 0.982 (**Figure 4B**). Both GSE26378 and GSE13904 datasets served as independent validation cohorts, achieving AUC, sensitivity, and specificity values of 0.970, 0.939, and 1.000, as well as 0.985, 0.923, and 1.000, respectively (**Figure 4C**, **Supplementary Figure 2A**). In parallel, the 13 features were used to develop another logistic regression model (13-gene signature) for distinguishing sepsis from ODs, which also demonstrated an AUC, sensitivity, and specificity of 1.000 in the training cohort (**Figure 4D**). In test cohort, the model achieved an AUC of 0.985, sensitivity of 0.923, and specificity of 1.000 (**Figure 4E**). The 13-gene signature showed optimal AUC values in two additional independent validation cohorts (**Figure 4F**, **Supplementary Figure 2B**). Furthermore, its performance in distinguishing sepsis from specific diseases is illustrated in **Supplementary Figure 3**. Finally, experimental validation using qRT-PCR demonstrated that the 28-gene signature could distinguish sepsis from controls with an AUC of 0.870 (**Figure 4G**), while the 13-gene signature discriminated sepsis from SIRS with an AUC of 0.745 (**Figure 4H**). The logistic regression models were supplied in **Supplementary Text**.

### Discovery of hub genes in sepsis

**Figure 5:**
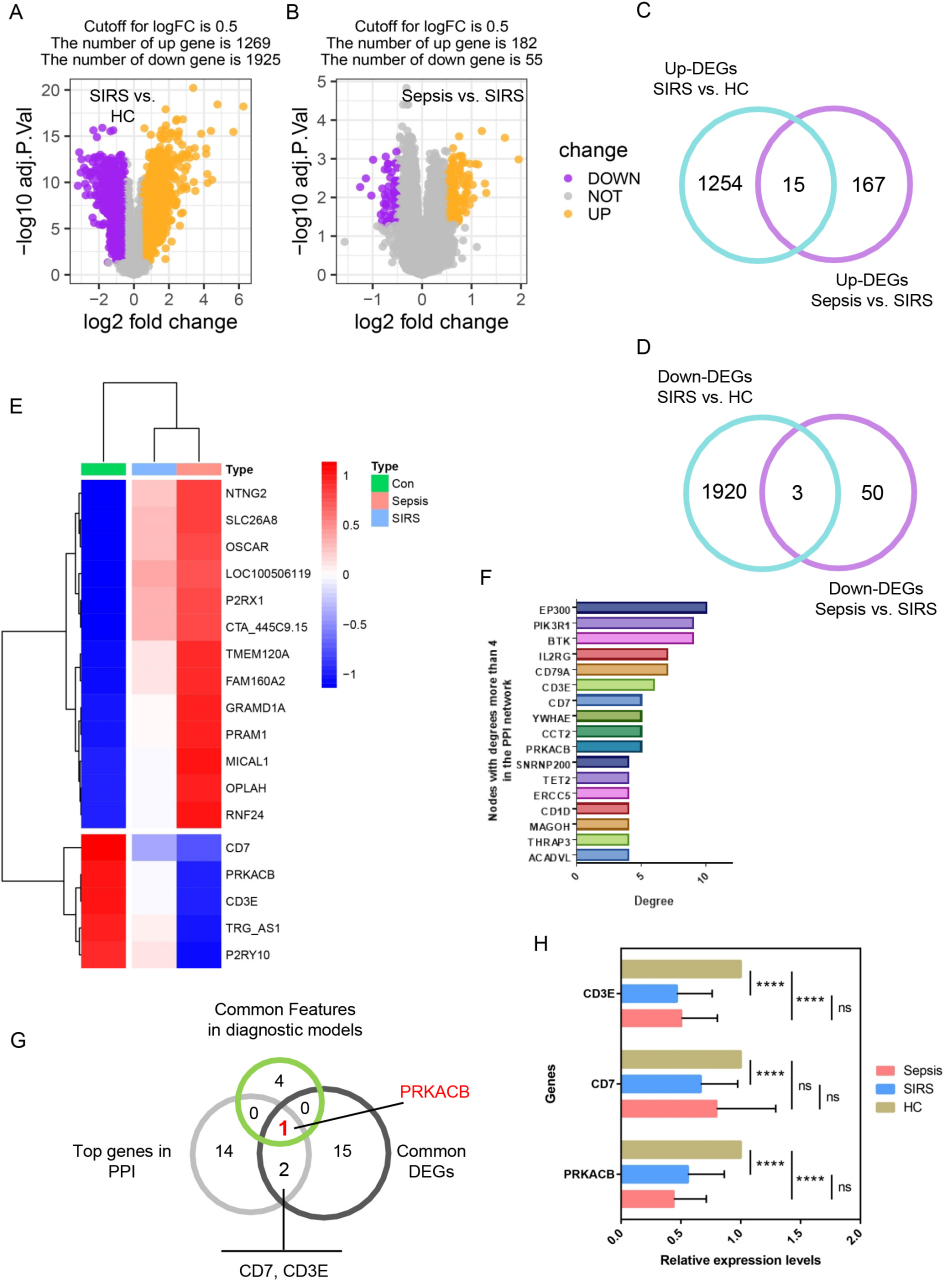
Identification of hub gene. **(A, B)** Difference analysis comparing SIRS with HC **(A)**, and sepsis with SIRS **(B)**. **(C)** Common up-regulated DEGs identified in both comparisons: SIRS vs. HC and sepsis vs. SIRS. **(D)** Common down-regulated DEGs shared by the two comparison groups. **(E)** Expression profiles of the common DEGs. **(F)** Top characteristics with a connectivity greater than 4 in the PPI network. **(G)** Identification of hub genes based on shared factors with high connectivity in the PPI network, common differential expression in both sepsis vs. SIRS and SIRS vs. HC, and the common features in both diagnostic models. **(H)** Validation of hub gene expression levels in whole blood samples using qRT-PCR. PPI, protein-protein interaction. **** indicates P < 0.0001.

To further explore the differences between SIRS and sepsis, we first log-transformed the dataset and adjusted the threshold for differential analysis to an adjusted p-value < 0.05 and |log_2_(foldchange)| > 0.5. This led to the identification of 1,269 upregulated and 1,925 downregulated genes in SIRS compared to HC (**Figure 5A**), and 182 upregulated and 55 downregulated genes in sepsis compared to SIRS (**Figure 5B**). Notably, 15 common upregulated genes and 3 common downregulated genes were shared between the two comparisons (**Figures 5C–E**). The expression profiles of these 18 genes were visualized in a heatmap (**Figure 5E**). Additionally, we constructed a PPI network for the DEGs, which included 114 edges and 95 nodes (**Supplementary Figure 4A**). Among the 17 nodes with a connectivity degree greater than 4 (**Figure 5F**), PRKACB was identified as a shared feature across the DEGs (**Figures 5A, B**) and the dual diagnostic models (**Figure 5G**). qRT-PCR validation confirmed that PRKACB was significantly downregulated in both sepsis and SIRS patients. Furthermore, a ceRNA network was developed to investigate the regulatory interactions of PRKACB (**Supplementary Figure 4B**).

### PRKACB with high expression in myeloid cells

**Figure 6:**
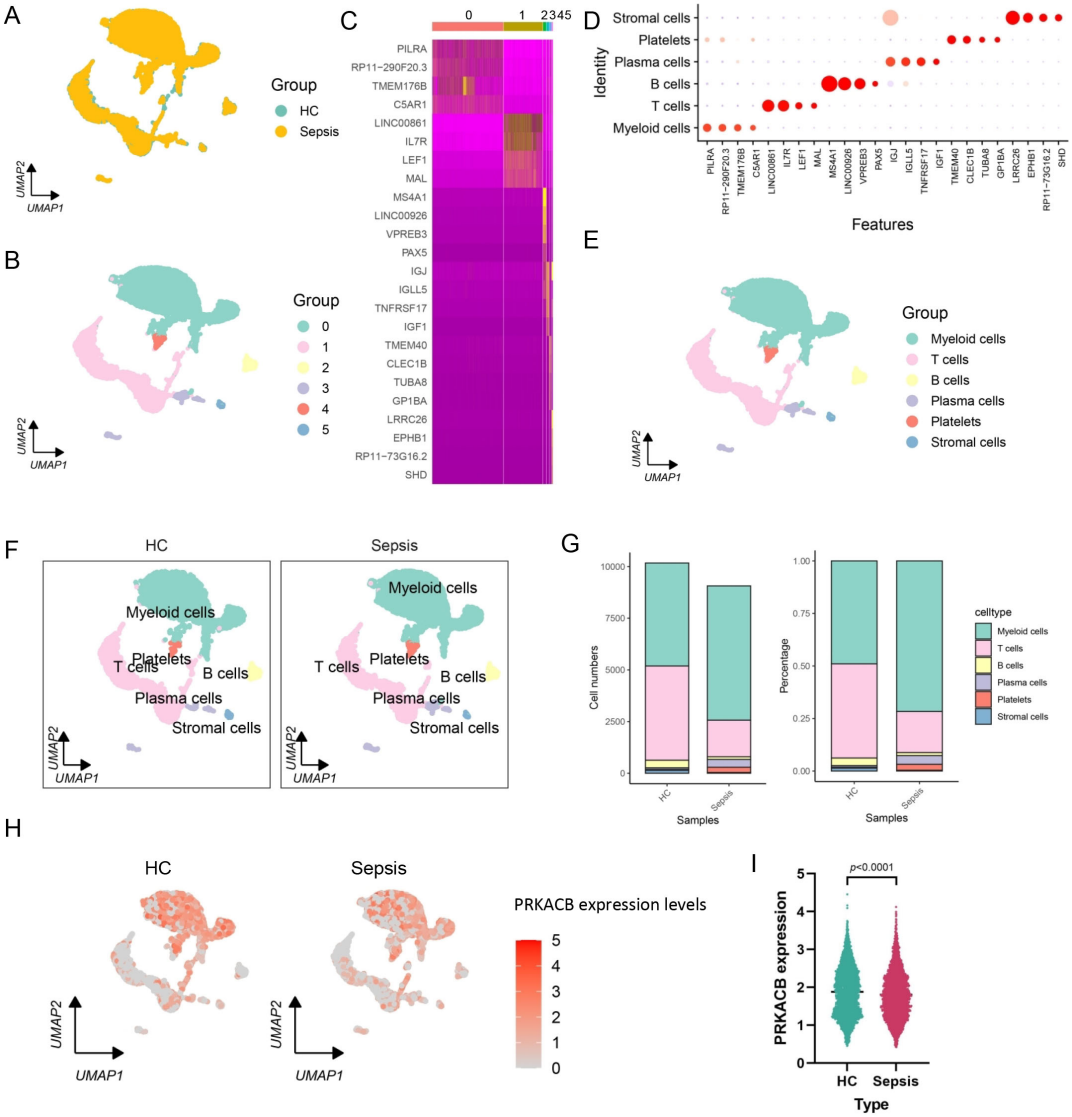
Single-cell RNA-sequencing analysis for sepsis. **(A)** UMAP dimensionality reduction analysis depicting the uniform distribution of samples from HC and sepsis. **(B)** Single-cell clustering at resolution = 0.1. **(C)** Heatmap showing the expression of characteristic genes for each cluster. **(D)** Dotplot depicting cell type annotations based on the characteristic genes of each cluster. **(E)** UMAP used to display the spatial distribution of annotated cell types. **(F)** Distribution differences of annotated cells between the HC and sepsis groups. **(G)** Relative abundance percentage of different cell types in the HC and sepsis groups. **(H, I)** PRKACB showing weak expression levels in myeloid cells from sepsis cases. Statistical comparisons were performed using per-sample cell-type proportions. Differences between HC and sepsis groups were assessed using the Wilcoxon rank-sum test, and exact P values are indicated in the figure. UMAP, Uniform Manifold Approximation and Projection.

**Figure 7:**
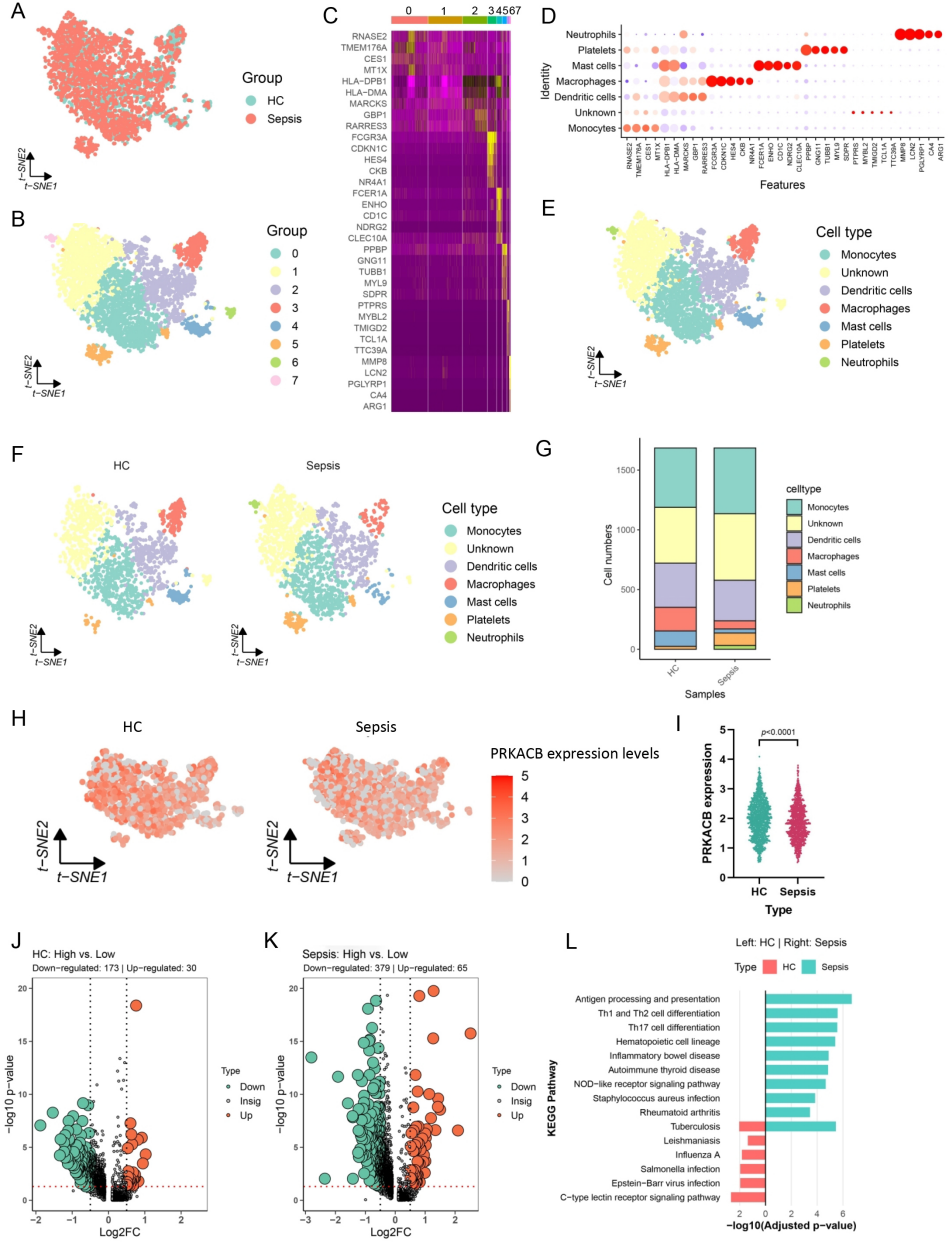
PRKACB expression on myeloid cells. **(A)** tSNE dimensionality reduction analysis depicting the uniform distribution of samples from HC and sepsis. **(B)** Single-cell clustering at resolution = 0.1. **(C)** Heatmap showing the expression of characteristic genes for each cluster. **(D)** Dotplot depicting cell type annotations based on the characteristic genes of each cluster. **(E)** tSNE used to display the spatial distribution of annotated cell types. **(F)** Distribution differences of annotated cells between the HC and sepsis groups. **(G)** Relative abundance percentage of different cell types in the HC and sepsis groups. **(H, I)** PRKACB showing weak expression levels in myeloid cells from sepsis cases. **(J)** Volcano plot showing differential analysis between PRKACB high- and low-expressed cells in the HC cases. PRKACB high- and low-expressed cells were classified based on the median expression value of PRKACB. **(K)** Differential analysis between PRKACB high- and low-expressed cells in the sepsis cases. **(L)** KEGG enrichment analysis of the DEGs involved in the underlying functions of PRKACB. Per-sample myeloid subtype composition shown as cell counts and proportions. Statistical comparisons were performed on per-sample cell-type proportions using the Wilcoxon rank-sum test, and exact P values are indicated. Differential expression for PRKACB-high versus PRKACB-low comparisons was performed using MAST with FDR-adjusted P < 0.05. tSNE, t-distributed Stochastic Neighbor Embedding.

Based on the results shown in **Figure 5**, PRKACB was identified as a target gene for sepsis. Subsequently, scRNA-sequencing was performed to analyze immune cell distribution, target gene expression patterns, and explore the potential functions of the target gene. UMAP clustering revealed that cells from different groups were evenly distributed, and could be preliminarily categorized into six clusters (resolution = 0.1) (**Figures 6A, B**). Using the characteristic genes for each cluster, the cell types were annotated as myeloid cells, T cells, B cells, plasma cells, platelets, and stromal cells (**Figures 6C–E**). We performed a comparative analysis of the relative abundance or percentage of each cell type, which revealed a significant increase in myeloid cells in sepsis patients, while T cells showed a significant decrease (**Figures 6F, G**). Furthermore, we observed that PRKACB was primarily expressed in the myeloid cell region and significantly downregulated in sepsis (**Figures 6H, I**). Thus, myeloid cells became the focus of our subsequent studies. We extracted the myeloid cell population for further analysis, which revealed that myeloid cells could be further divided into monocytes, dendritic cells, macrophages, mast cells, neutrophils, and platelets (**Figures 7A–E**). After ensuring an equal number of cells from the HC and sepsis groups, we found that macrophages were significantly downregulated in sepsis, while neutrophils were significantly upregulated (**Figures 7F, G**). Additionally, PRKACB expression was significantly lower in the myeloid cells of the sepsis group (**Figures 7H, I**), which is consistent with the previous results (**Figures 6H, I**).

### PRKACB associated with cell viability in macrophages

**Figure 8:**
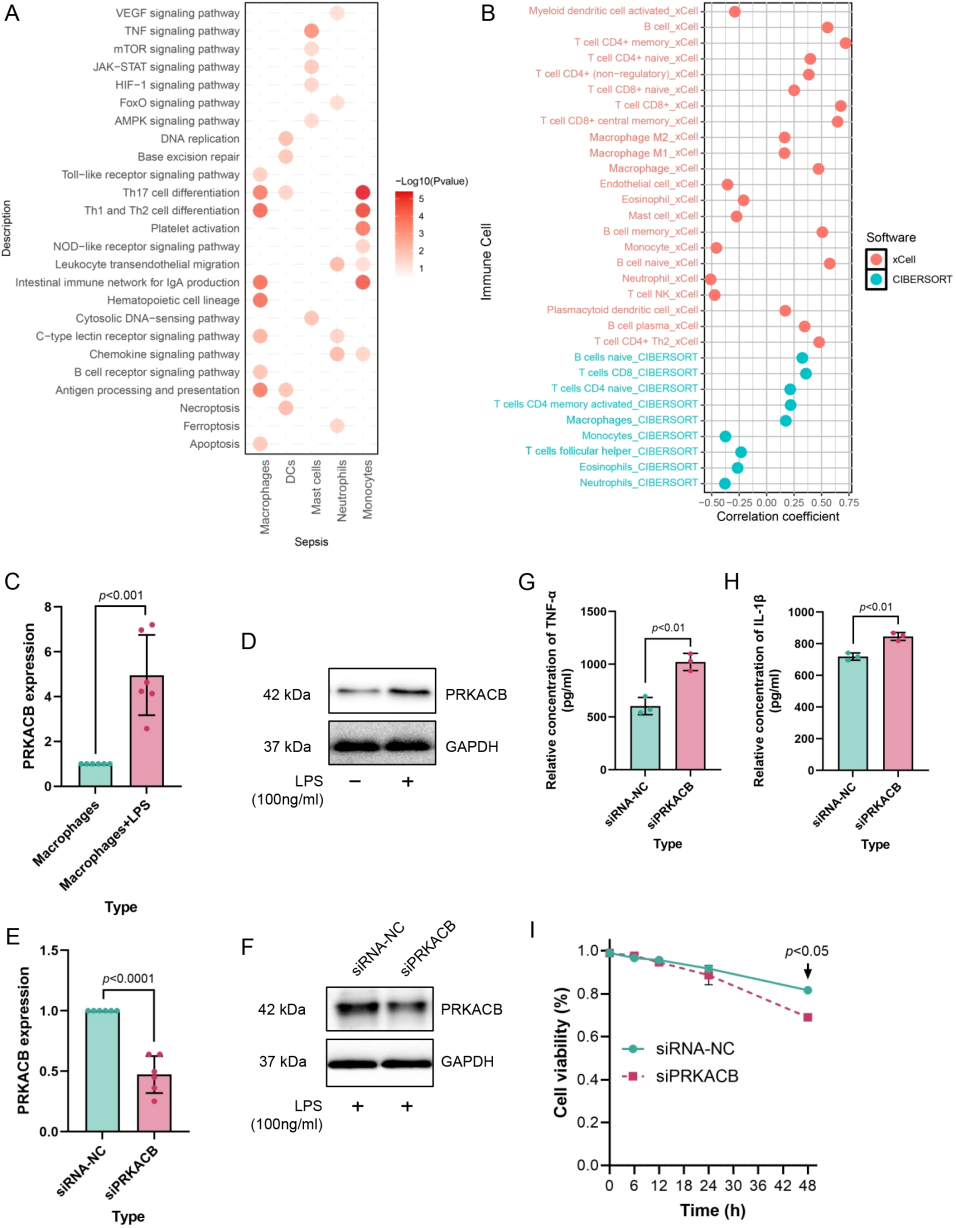
Functional analysis of PRKACB. **(A)** Dotplot depicting the functions associated with PRKACB in different myeloid cells based on scRNA sequencing analysis and KEGG enrichment analysis. **(B)** Scatterplot showing the association of PRKACB with immune cells based on xCELL and CIBERSORT platforms. **(C, D)** PRKACB shows significantly higher expression in THP-1 macrophages treated with 100 ng/ml LPS, as measured by qRT-PCR **(C)** and western blotting **(D)**. **(E, F)** After silencing the target gene in THP-1 cells using siRNA, PRKACB exhibits weak expression levels, both at the gene level **(E)** and protein level **(F)**. **(G, H)** siPRKACB significantly promoted the release of TNF-alpha **(G)** and IL-1beta **(H)** cytokines in macrophages pretreated with LPS. **(I)** Cell viability assessment using the CCK-8 kit, indicating that siPRKACB is detrimental to maintaining cell viability.

To further investigate the molecular function of PRKACB, we divided cells from the HC and sepsis groups into PRKACB high expression and low expression groups based on the median expression level of PRKACB and identified DEGs (**Figures 7J, K**). We then analyzed the enriched KEGG pathways of these DEGs to hypothesize the functions of PRKACB in myeloid cells. The results showed that in sepsis, PRKACB was involved in antigen processing and presentation, Th1 and Th2 cell differentiation, Th17 cell differentiation, NOD-like receptor signaling pathway, and other processes, with PRKACB being more related to infectious diseases in HC (**Figure 7L**). In our subsequent studies, we focused more on the function of PRKACB in myeloid cells from sepsis. The results indicated that in macrophages, PRKACB may be involved in antigen processing and presentation, Th1 and Th2 cell differentiation, Th17 cell differentiation, and apoptosis; in monocytes, PRKACB may participate in the NOD-like receptor signaling pathway; in neutrophils, PRKACB may be involved in the FoxO signaling pathway and ferroptosis (**Figure 8A**). Additionally, immune cell infiltration analysis using the CIBERSORT and xCell platforms revealed a strong correlation between PRKACB expression levels and the abundance of macrophages and neutrophils (**Figure 8B**). From our previous analysis, we observed significant changes in the proportion of macrophages and neutrophils in sepsis (**Figures 7F, G**). However, based on the actual content and function of macrophages in real samples, we focused on PRKACB function analysis specifically in macrophages.

Mechanistically, PRKACB encodes the catalytic subunit β of protein kinase A (PKA), a core effector of the cAMP–PKA axis that is widely recognized to modulate inflammatory signaling and stress adaptation in myeloid cells. Based on our single-cell localization of PRKACB to the myeloid compartment and the pathway enrichment signals involving immune activation and cell-death–related processes, we hypothesized that reduced PRKACB may weaken PKA-dependent homeostatic restraint under inflammatory stimulation, thereby shifting macrophages toward a hyper-inflammatory state and/or compromised viability. Therefore, we selected an LPS-driven THP-1 macrophage model as a tractable system to functionally probe whether PRKACB downregulation alters canonical pro-inflammatory cytokine output (TNF-α and IL-1β) and cell viability under inflammatory stress. The results showed that PRKACB was highly expressed in LPS-activated THP-1 macrophages (**Figures 8C, D**), and siRNA effectively reduced PRKACB expression levels (**Figures 8E, F**). Furthermore, we found that after treating THP-1 cells with 100 ng/ml LPS for 48 hours, the low expression of PRKACB enhanced the release of TNF-α and IL-1β cytokines (**Figures 8G, H**), but significantly decreased THP-1 cell viability (**Figure 8I**). Based on these results, we hypothesize that PRKACB helps maintain macrophage cell viability, but when PRKACB expression is blocked, it may promote cell apoptosis by accelerating the release of TNF-α and other cytokines. This hypothesis will be further explored in future studies.

## Discussion

Sepsis syndromes have been recognized since ancient times but continue to pose significant challenges to modern medicine. The primary difficulties, on one hand, stem from the heterogeneity of triggers, diverse clinical manifestations, and the rapidly progressive and lethal nature of the condition. Therefore, there is an urgent need for biomarkers that can quickly and accurately detect the onset of sepsis. On the other hand, sepsis lacks effective treatment options or therapeutic targets. In this study, we employed the SVM-RFE machine learning algorithm and logistic regression to develop two diagnostic models for sepsis (i.e., a 28-gene signature and a 13-gene signature). We would also like to point out that our initial plan was to use a retrospective cohort to establish a prognostic model to predict the onset of sepsis. However, such sepsis cohorts are rare in the GEO database. We are currently working on establishing a prospective cohort to address this issue. Furthermore, based on the components of the diagnostic models, as well as subsequent single-cell analysis and molecular biology experiments, we selected PRKACB as the hub gene for this study.

In this study, PRKACB was prioritized as the core target for functional validation because it was consistently downregulated across all datasets, appeared in both diagnostic models, and showed the highest network connectivity among overlapping differentially expressed genes. Single-cell RNA sequencing further indicated that PRKACB is predominantly expressed in myeloid cells, particularly macrophages and neutrophils, which displayed the most significant compositional changes in sepsis. Transcriptional diagnostics of sepsis focuses on blood leukocyte genomics as a tool to classify patients into more homogeneous sub-groups for effective stratification (19). Biomarkers, including transcriptomic signatures, can distinguish critically ill patients with or without infection and differentiate between various pathogens based on host response signatures (20). SeptiCyte, an FDA-approved biomarker developed by Immunexpress, has shown the ability to differentiate ICU patients with infection-negative sepsis from those with infection-positive sepsis (21–23). Quantitative PCR analysis of four genes (CEACAM4, LAMP1, PLA2G7 and PLAC8) demonstrated significant differential expression, achieving an AUC of 0.95 (21). In another study, the differential expression of 11 genes from publicly available gene expression data yielded an AUC of 0.87 to distinguish sterile inflammation from sepsis, validated across 15 independent cohorts (24). Additionally, Bai H et al. identified BMP9 as a potential sepsis biomarker, found at low concentrations in patients and involved in macrophage recruitment (25). The SPINK1 protein level was significantly elevated in sepsis patients, achieving an AUC of 0.9096 in distinguishing sepsis from healthy controls (26), and combined with procalcitonin, SPINK1 yielded an AUC of 1.000. Furthermore, a neural network based on 29 preselected host mRNAs has been developed to diagnose infections, differentiate between bacterial and viral infections, and assess the risk of 30-day mortality (27). However, these biomarkers face limitations; they often lack clinical validation using whole blood or other clinical samples and may lack specificity in diagnosing sepsis, as they do not adequately differentiate sepsis from other pulmonary diseases. To benchmark our model against existing sepsis biomarkers, we compared CRP, PCT (CALCA), and IL-6 within the same datasets. As shown in **Supplementary Figure 5**, these classical markers displayed only moderate diagnostic accuracy (AUC 0.52–0.74), whereas our integrated multi-gene signature achieved higher and more stable discrimination, underscoring its potential clinical advantage over conventional biomarkers.

The present work provides an overview of the immune functions and ceRNA networks associated with PRKACB. The protein kinase CAMP-activated catalytic subunit beta (PRKACB) is an important downstream effector molecule of the cAMP/PKA signaling pathway and participates in the regulation of various cellular processes. Previous studies have reported that PRKACB contributes to cell proliferation in acute myeloid leukemia and inhibits apoptosis by functioning as a target of miR-496 (28–30). In the ceRNA network of this study, we identified several miRNAs, including let-7 family microRNAs (hsa-let-7a-3p, hsa-let-7b-3p, hsa-let-7d-3p, and hsa-let-7f-3p), hsa-miR-1228-3p, hsa-miR-130a-3p, and hsa-miR-129-5p, which can target PRKACB (**Supplementary Figure 4B**). Notably, hsa-let-7a has been shown to contribute to cell cycle arrest at the sub-G1 phase in HCT-116 colorectal cancer cells, and cell cycle arrest is closely associated with apoptosis (31). hsa-let-7b-3p has also been implicated in the progression of osteoarthritis by modulating interleukin-1β (IL-1β) effects and subsequent apoptosis (32). Moreover, Yin et al. reported that hsa-miR-130a-3p inhibits cellular growth in intestinal epithelial cells (33). It is also involved in coronary atherosclerosis and coronary artery disease, primarily due to the regulation of TRPM3 mRNA as a target of hsa-miR-130a-3p, which controls the proliferation and contractility of vascular smooth muscle cells (34). These previous findings align with our study’s conclusions: PRKACB in macrophages is closely related to biological processes such as apoptosis, which further supports the validity of our analysis.

PRKACB was also identified as a key gene in sepsis in the study by Zeng et al., which utilized bioinformatics analysis on four GEO datasets (35). It was found to be significantly downregulated in sepsis and suggested to promote sepsis progression via the MAPK signaling pathway (35, 36). Additionally, PRKACB plays essential roles in osteoarthritis development, particularly in immune cells, especially macrophages (37), although the specific molecular mechanisms remain unclear. Tanaka et al. proposed that PRKACA/PRKACB fusions might be key to its functional role (38). Furthermore, Newman et al. concluded that PRKACB is a novel, differentially methylated region-associated marsupial imprinted gene (39), which might indicate a conserved selective pressure for imprinting of the protein kinase A (PKA) signaling pathway in therians, with the two lineages adapting by imprinting different genes. Espiard et al. also reported that PRKACB variants in skeletal disease or adrenocortical hyperplasia primarily affect PKA signaling (40). Variants such as p.K286del and c.899C>T in the PRKACB gene are highly conserved. The p.K286del variant affects protein stability and enhances PKA signaling, which is linked to various defects and neoplasms (40). Furthermore, mutations in PRKAR1A are associated with impaired cAMP-dependent signaling (40). However, to date, the function of PRKACB in macrophages has not been fully elucidated.

Zhao et al. concluded that ADAR1 protects pulmonary macrophages against pyroptosis in sepsis via the miR-21/A20/NLRP3 axis (41). It is well-known that peripheral immune cells undergo phenomena such as apoptosis, necroptosis, and pyroptosis as they combat pathogen invasion during the early stages of sepsis. Among these, macrophages and neutrophils, as the primary line of defense, exhibit the most severe cell death during the initial phase of sepsis. Macrophages recognize and clear pathogens in response to signals released by infected cells, while neutrophils promote macrophage pyroptosis through the formation of extracellular traps, influencing the inflammatory response (42, 43). Multiple cell death pathways, including necrosis, apoptosis, necroptosis, NETosis, pyroptosis, and autophagy-induced cell death, can be activated as sepsis progresses (44). However, to date, research on macrophages in sepsis has primarily focused on pyroptosis rather than apoptosis. Apoptosis plays a pivotal role in sepsis-induced immune dysregulation (45, 46). Malireddi et al. also indicated that targeting apoptosis could be a potential treatment strategy for sepsis (47). However, microbial products and host-derived factors can delay neutrophil apoptosis during sepsis, largely through a process dependent on the anti-apoptotic protein myeloid cell leukemia 1 (MCL1) (48, 49). Therefore, in our future studies, we aim to focus more on the role of macrophage apoptosis in the sepsis process and investigate the function and mechanisms of PRKACB in macrophage apoptosis.

In addition to macrophages, PRKACB expression also showed a correlation with neutrophil abundance in sepsis. Given the essential role of neutrophils in early inflammatory responses and macrophage–neutrophil crosstalk during sepsis, future studies will investigate whether PRKACB contributes to neutrophil activation or intercellular signaling within the myeloid compartment.

The present work has several limitations that need to be addressed. First, the current analysis is based on retrospective GEO datasets rather than prospective clinical cohorts, which limits the evaluation of the diagnostic model’s prognostic performance and its generalizability to real-world settings. Future work will establish a prospective, multicenter cohort to validate and optimize the diagnostic model by incorporating clinical outcomes and improving its translational applicability. Second, while we provide initial functional support in an LPS-stimulated THP-1 macrophage model, the downstream signaling mechanisms and *in vivo* relevance of PRKACB in sepsis remain to be established, including validation in primary human myeloid cells and animal models. We recognize this as an important limitation and have initiated follow-up studies to investigate PRKACB-dependent regulation of macrophage polarization, apoptosis, and mitochondrial function. Additionally, the diagnostic model for sepsis did not perform optimally during qRT-PCR validation, likely due to the limited clinical sample size, and the model may require further optimization and refinement. Finally, while the 13-gene signature was intended to distinguish sepsis from other diseases, the validation phase focused solely on its ability to differentiate between sepsis and SIRS, without assessing its performance against other conditions. Addressing these limitations will be crucial for enhancing the robustness and clinical applicability of the findings. In parallel, clinical translation of the 28- and 13-gene signatures will be advanced through prospective multicenter validation with pre-specified cutoffs, evaluation of assay turnaround time and scalability for a targeted qPCR panel, and head-to-head benchmarking against conventional biomarkers such as CRP, PCT, and IL-6 in clinically overlapping inflammatory conditions.

## Conclusion

In this study, we developed 28- and 13-gene signatures for discriminating sepsis from healthy controls and from clinically overlapping inflammatory conditions. Additionally, multi-layer analyses identified PRKACB as a downregulated myeloid hub gene, and macrophage knockdown supported its association with increased inflammatory cytokine output and reduced viability. PRKACB merits further mechanistic, *in vivo*, and prospective evaluation

## Data Availability

All datasets analyzed in this study are publicly available in the Gene Expression Omnibus (GEO) repository under the following accession numbers: GSE13904, GSE26050, GSE42834, GSE45291, GSE84844, GSE26378, GSE72326, GSE66099, and GSE175453 (https://www.ncbi.nlm.nih.gov/geo/). The processed data supporting the findings of this study are included in the article and its **Supplementary Materials**. Further inquiries can be directed to the corresponding author.

## References

[1] Evans L Rhodes A Alhazzani W Antonelli M Coopersmith CM French C . Surviving sepsis campaign: international guidelines for management of sepsis and septic shock 2021. Intensive Care Med. (2021) 47:1181–247. doi: 10.1007/s00134-021-06506-y, PMID: 34599691 PMC8486643

[2] Costello C Davies FE Cook G Vela-Ojeda J Omel J Rifkin RM . INSIGHT MM: a large, global, prospective, non-interventional, real-world study of patients with multiple myeloma. Future Oncol. (2019) 15:1411–28. doi: 10.2217/fon-2019-0013, PMID: 30816809 PMC6854441

[3] Wang Q Wang C Zhang W Tao Y Guo J Liu Y . Identification of biomarkers related to sepsis diagnosis based on bioinformatics and machine learning and experimental verification. Front Immunol. (2023) 14:1087691. doi: 10.3389/fimmu.2023.1087691, PMID: 37449204 PMC10337583

[4] Cohen J Vincent JL Adhikari NK MaChado FR Angus DC Calandra T . Sepsis: a roadmap for future research. Lancet Infect Dis. (2015) 15:581–614. doi: 10.1016/S1473-3099(15)70112-X, PMID: 25932591

[5] Kuang L Wu Y Shu J Yang J Zhou H Huang X . Pyroptotic Macrophage-Derived Microvesicles Accelerate Formation of Neutrophil Extracellular Traps via GSDMD-N-expressing Mitochondrial Transfer during Sepsis. Int J Biol Sci. (2024) 20:733–50. doi: 10.7150/ijbs.87646, PMID: 38169726 PMC10758106

[6] She H Tan L Du Y Zhou Y Guo N Zhang J . VDAC2 malonylation participates in sepsis-induced myocardial dysfunction via mitochondrial-related ferroptosis. Int J Biol Sci. (2023) 19:3143–58. doi: 10.7150/ijbs.84613, PMID: 37416771 PMC10321281

[7] Mirijello A Tosoni A . On behalf of the internal medicine sepsis study group. New Strategies for Treatment of Sepsis. Med (Kaunas). (2020) 56:527. doi: 10.3390/medicina56100527, PMID: 33050538 PMC7599752

[8] Zampieri FG Bagshaw SM Semler MW . Fluid therapy for critically ill adults with sepsis: A review. JAMA. (2023) 329:1967–80. doi: 10.1001/jama.2023.7560, PMID: 37314271

[9] Qiu X Lei YP Zhou RX . SIRS, SOFA, qSOFA, and NEWS in the diagnosis of sepsis and prediction of adverse outcomes: a systematic review and meta-analysis. Expert Rev Anti Infect Ther. (2023) 21:891–900. doi: 10.1080/14787210.2023.2237192, PMID: 37450490

[10] Arroyo MG Slovis NM Moore GE Taylor SD . Factors associated with survival in 97 horses with septic pleuropneumonia. J Vet Intern Med. (2017) 31:894–900. doi: 10.1111/jvim.14679, PMID: 28271546 PMC5435057

[11] Sheats MK . A comparative review of equine SIRS, sepsis, and neutrophils. Front Vet Sci. (2019) 6:69. doi: 10.3389/fvets.2019.00069, PMID: 30931316 PMC6424004

[12] Castellheim A Brekke OL Espevik T Harboe M Mollnes TE . Innate immune responses to danger signals in systemic inflammatory response syndrome and sepsis. Scand J Immunol. (2009) 69:479–91. doi: 10.1111/j.1365-3083.2009.02255.x, PMID: 19439008

[13] Zhang WY Chen ZH An XX Li H Zhang HL Wu SJ . Analysis and validation of diagnostic biomarkers and immune cell infiltration characteristics in pediatric sepsis by integrating bioinformatics and machine learning. World J Pediatr. (2023) 19:1094–103. doi: 10.1007/s12519-023-00717-7, PMID: 37115484 PMC10533616

[14] Huang YH Chen CJ Shao SC Li CH Hsiao CH Niu KY . Comparison of the diagnostic accuracies of monocyte distribution width, procalcitonin, and C-reactive protein for sepsis: A systematic review and meta-analysis. Crit Care Med. (2023) 51:e106–14. doi: 10.1097/CCM.0000000000005820, PMID: 36877030 PMC10090344

[15] Medina-Ortiz D Khalifeh A Anvari-Kazemabad H Davari MD . Interpretable and explainable predictive machine learning models for data-driven protein engineering. Biotechnol Adv. (2025) 79:108495. doi: 10.1016/j.bioteChadv.2024.108495, PMID: 39645211

[16] Gao XJ Ciura K Ma Y Mikolajczyk A Jagiello K Wan Y . Toward the integration of machine learning and molecular modeling for designing drug delivery nanocarriers. Adv Mater. (2024) 36:e2407793. doi: 10.1002/adma.202407793, PMID: 39252670

[17] Li Z Yu B Qi F Li F . KIF11 serves as an independent prognostic factor and therapeutic target for patients with lung adenocarcinoma. Front Oncol. (2021) 11:670218. doi: 10.3389/fonc.2021.670218, PMID: 33968780 PMC8103954

[18] Corley KT Donaldson LL Furr MO . Arterial lactate concentration, hospital survival, sepsis and SIRS in critically ill neonatal foals. Equine Vet J. (2005) 37:53–9. doi: 10.2746/0425164054406856, PMID: 15651735

[19] Saxena J Das S Kumar A Sharma A Sharma L Kaushik S . Biomarkers in sepsis. Clin Chim Acta. (2024) 562:119891. doi: 10.1016/j.cca.2024.119891, PMID: 39067500

[20] van Engelen TSR Wiersinga WJ Scicluna BP van der Poll T . Biomarkers in sepsis. Crit Care Clin. (2018) 34:139–52. doi: 10.1016/j.ccc.2017.08.010, PMID: 29149935

[21] McHugh L Seldon TA Brandon RA Kirk JT Rapisarda A Sutherland AJ . A molecular host response assay to discriminate between sepsis and infection-negative systemic inflammation in critically ill patients: discovery and validation in independent cohorts. PLoS Med. (2015) 12:e1001916. doi: 10.1371/journal.pmed.1001916, PMID: 26645559 PMC4672921

[22] Verboom DM Koster-Brouwer ME Ruurda JP van Hillegersberg R van Berge Henegouwen MI Gisbertz SS . A pilot study of a novel molecular host response assay to diagnose infection in patients after high-risk gastro-intestinal surgery. J Crit Care. (2019) 54:83–7. doi: 10.1016/j.jcrc.2019.07.020, PMID: 31398685

[23] Denny KJ Lea RA Lindell-Innes R Haupt LM Heffernan AJ Harvey NR . Diagnosing sepsis in the ICU: Comparison of a gene expression signature to pre-existing biomarkers. J Crit Care. (2023) 76:154286. doi: 10.1016/j.jcrc.2023.154286, PMID: 36965223

[24] Sweeney TE Shidham A Wong HR Khatri P . A comprehensive time-course-based multicohort analysis of sepsis and sterile inflammation reveals a robust diagnostic gene set. Sci Transl Med. (2015) 7:287ra71. doi: 10.1126/scitranslmed.aaa5993, PMID: 25972003 PMC4734362

[25] Bai H Lu Q Wu C Xu F Liu J Wang K . Bone morphogenetic protein 9 is a candidate prognostic biomarker and host-directed therapy target for sepsis. Sci Transl Med. (2024) 16:eadi3275. doi: 10.1126/scitranslmed.adi3275, PMID: 38295185

[26] Chen D Shi Z Gao X Yang Y Lei X Hu Y . SPINK1 is a potential diagnostic and prognostic biomarker for sepsis. Infect Drug Resist. (2024) 17:875–84. doi: 10.2147/IDR.S440117, PMID: 38476769 PMC10929552

[27] Mayhew MB Buturovic L Luethy R Midic U Moore AR Roque JA . A generalizable 29-mRNA neural-network classifier for acute bacterial and viral infections. Nat Commun. (2020) 11:1177. doi: 10.1038/s41467-020-14975-w, PMID: 32132525 PMC7055276

[28] Wang Y Guo T Liu Q Xie X . CircRAD18 accelerates the progression of acute myeloid leukemia by modulation of miR-206/PRKACB axis. Cancer Manag Res. (2020) 12:10887–96. doi: 10.2147/CMAR.S277432, PMID: 33154668 PMC7608482

[29] Skalhegg BS Tasken K . Specificity in the cAMP/PKA signaling pathway. Differential expression, regulation, and subcellular localization of subunits of PKA. Front Biosci. (2000) 5:D678–693. doi: 10.2741/a195, PMID: 10922298

[30] Wu D-M Wen X Han X-R Wang S Wang YJ Shen M . Role of circular RNA DLEU2 in human acute myeloid leukemia. Mol Cell Biol. (2018) 38:e00259–18. doi: 10.1128/MCB.00259-18, PMID: 30037980 PMC6168983

[31] Mozammel N Baghbani E Amini M Jodeiry Zaer S Baghay Esfandyari Y Tohidast M . The Simultaneous Effects of miR-145-5p and hsa-let-7a-3p on Colorectal Tumorigenesis: *In Vitro* Evidence. Adv Pharm Bull. (2024) 14:231–40. doi: 10.34172/apb.2024.004, PMID: 38585468 PMC10997926

[32] Tao SC Huang JY Gao Y Li ZX Wei ZY Dawes H . Small extracellular vesicles in combination with sleep-related circRNA3503: A targeted therapeutic agent with injectable thermosensitive hydrogel to prevent osteoarthritis. Bioact Mater. (2021) 6:4455–69. doi: 10.1016/j.bioactmat.2021.04.031, PMID: 34027234 PMC8120802

[33] Yin J Ye YL Hu T Xu LJ Zhang LP Ji RN . Hsa_circRNA_102610 upregulation in Crohn’s disease promotes transforming growth factor-β1-induced epithelial-mesenchymal transition *via* sponging of hsa-miR-130a-3p. World J Gastroenterol. (2020) 26:3034–55. doi: 10.3748/wjg.v26.i22.3034, PMID: 32587447 PMC7304108

[34] Pan RY Liu P Zhou HT Sun WX Song J Shu J . Circular RNAs promote TRPM3 expression by inhibiting hsa-miR-130a-3p in coronary artery disease patients. Oncotarget. (2017) 8:60280–90. doi: 10.18632/oncotarget.19941, PMID: 28947970 PMC5601138

[35] Zeng X Feng J Yang Y Zhao R Yu Q Qin H . Screening of key genes of sepsis and septic shock using bioinformatics analysis. J Inflammation Res. (2021) 14:829–41. doi: 10.2147/JIR.S301663, PMID: 33737824 PMC7962593

[36] Thompson ED . Neoplastic progression in macroscopic precursor lesions of the pancreas. Arch Pathol Lab Med. (2024) 148:980–8. doi: 10.5858/arpa.2023-0358-RA, PMID: 38386006

[37] Zhao C . Identifying the hub gene and immune infiltration of osteoarthritis by bioinformatical methods. Clin Rheumatol. (2021) 40:1027–37. doi: 10.1007/s10067-020-05311-0, PMID: 32785809

[38] Tanaka M Takeshita K Kunita A Hasegawa K Ushiku T . PRKACA/PRKACB fusions in pancreatobiliary intraductal oncocytic papillary neoplasms including those with atypical morphology: an analysis of 22 cases expanding morphologic spectrum. Am J Surg Pathol. (2024) 48:1032–40. doi: 10.1097/PAS.0000000000002259, PMID: 38841868

[39] Newman T Bond DM Ishihara T Rizzoli P Gouil Q Hore TA . PRKACB is a novel imprinted gene in marsupials. Epigenet Chromatin. (2024) 17:29. doi: 10.1186/s13072-024-00552-8, PMID: 39342354 PMC11438212

[40] Espiard S Drougat L Settas N Haydar S Bathon K London E . PRKACB variants in skeletal disease or adrenocortical hyperplasia: effects on protein kinase A. Endocr Relat Cancer. (2020) 27:647–56. doi: 10.1530/ERC-20-0309, PMID: 33055300 PMC8728871

[41] Zhao X Xie J Duan C Wang L Si Y Liu S . ADAR1 protects pulmonary macrophages from sepsis-induced pyroptosis and lung injury through miR-21/A20 signaling. Int J Biol Sci. (2024) 20:464–85. doi: 10.7150/ijbs.86424, PMID: 38169584 PMC10758098

[42] Narasimhan PB Marcovecchio P Hamers AAJ Hedrick CC . Nonclassical monocytes in health and disease. Annu Rev Immunol. (2019) 37:439–56. doi: 10.1146/annurev-immunol-042617-053119, PMID: 31026415

[43] Chen L Zhao Y Lai D Zhang P Yang Y Li Y . Neutrophil extracellular traps promote macrophage pyroptosis in sepsis. Cell Death Dis. (2018) 9:597. doi: 10.1038/s41419-018-0538-5, PMID: 29789550 PMC5964241

[44] Lelubre C Vincent JL . Mechanisms and treatment of organ failure in sepsis. Nat Rev Nephrol. (2018) 14:417–27. doi: 10.1038/s41581-018-0005-7, PMID: 29691495

[45] Hotchkiss RS Monneret G Payen D . Sepsis-induced immunosuppression: from cellular dysfunctions to immunotherapy. Nat Rev Immunol. (2013) 13:862–74. doi: 10.1038/nri3552, PMID: 24232462 PMC4077177

[46] Girardot T Rimmelé T Venet F Monneret G . Apoptosis-induced lymphopenia in sepsis and other severe injuries. Apoptosis. (2017) 22:295–305. doi: 10.1007/s10495-016-1325-3, PMID: 27812767

[47] Malireddi RKS Gurung P Kesavardhana S Samir P Burton A Mummareddy H . Innate immune priming in the absence of TAK1 drives RIPK1 kinase activity-independent pyroptosis, apoptosis, necroptosis, and inflammatory disease. J Exp Med. (2020) 217:jem.20191644. doi: 10.1084/jem.20191644, PMID: 31869420 PMC7062518

[48] Taneja R Parodo J Jia SH Kapus A Rotstein OD Marshall JC . Delayed neutrophil apoptosis in sepsis is associated with maintenance of mitochondrial transmembrane potential and reduced caspase-9 activity. Crit Care Med. (2004) 32:1460–9. doi: 10.1097/01.ccm.0000129975.26905.77, PMID: 15241089

[49] Murphy MP Caraher E . Mcl-1 is vital for neutrophil survival. Immunol Res. (2015) 62:225–33. doi: 10.1007/s12026-015-8655-z, PMID: 25929430

