# Supplementary material for: Multi-omics machine learning identifies diagnostic gene signatures and functionally supports PRKACB involvement in macrophage inflammatory responses in sepsis
Source: Front Immunol. 2026 Jan 15;16:1611348. doi: 10.3389/fimmu.2025.1611348 (PMC12852012; doi:10.3389/fimmu.2025.1611348)
Supplement: Supplementary file 2 [file Table2.docx]

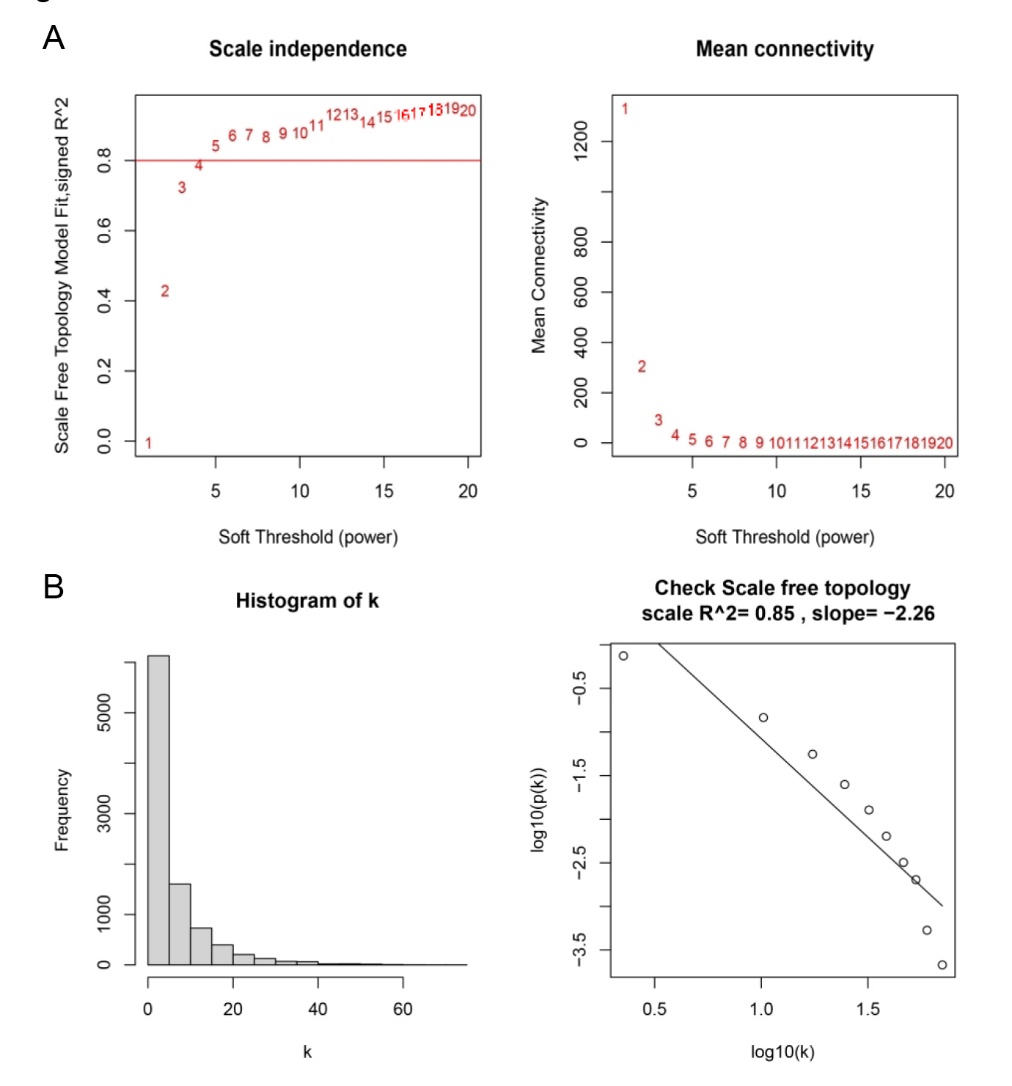


Supplementary Figure 1. Weighted gene co-expression network analysis. (A) The scale-free topology model used to identify the optimal β value. β = 5 was selected as the soft threshold based on average connectivity and scale independence. (B) Histogram of connectivity distribution (left) and scatter plots (right) evaluating the scale-free topology at β = 5.


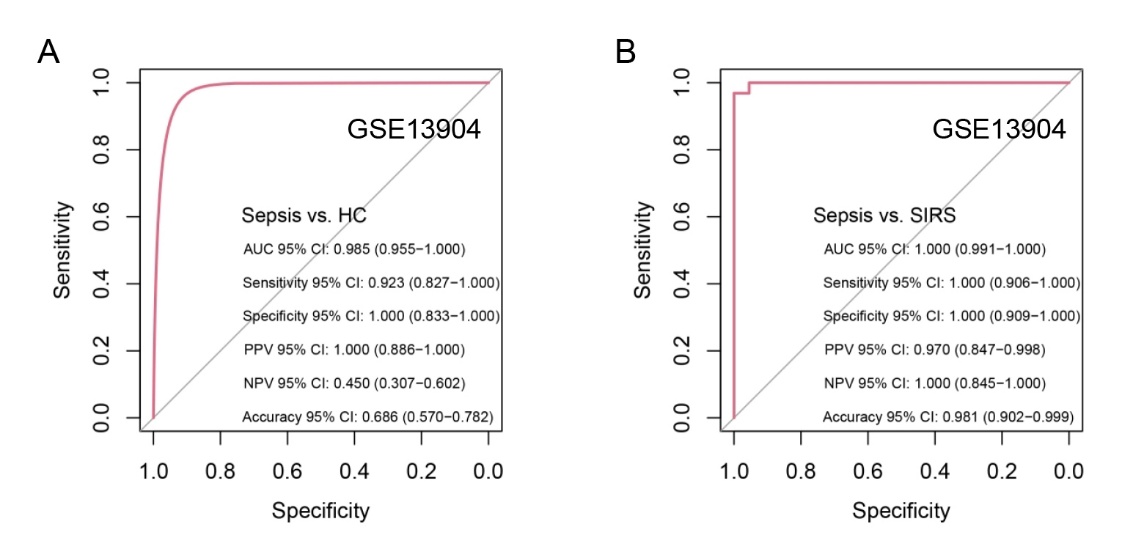


Supplementary Figure 2. Diagnostic Performance of Signatures in the GSE13904 Dataset. (A,B) Evaluation of the diagnostic performance of the 28-gene (A) and 13-gene (B) signatures for sepsis in the GSE13904 dataset.


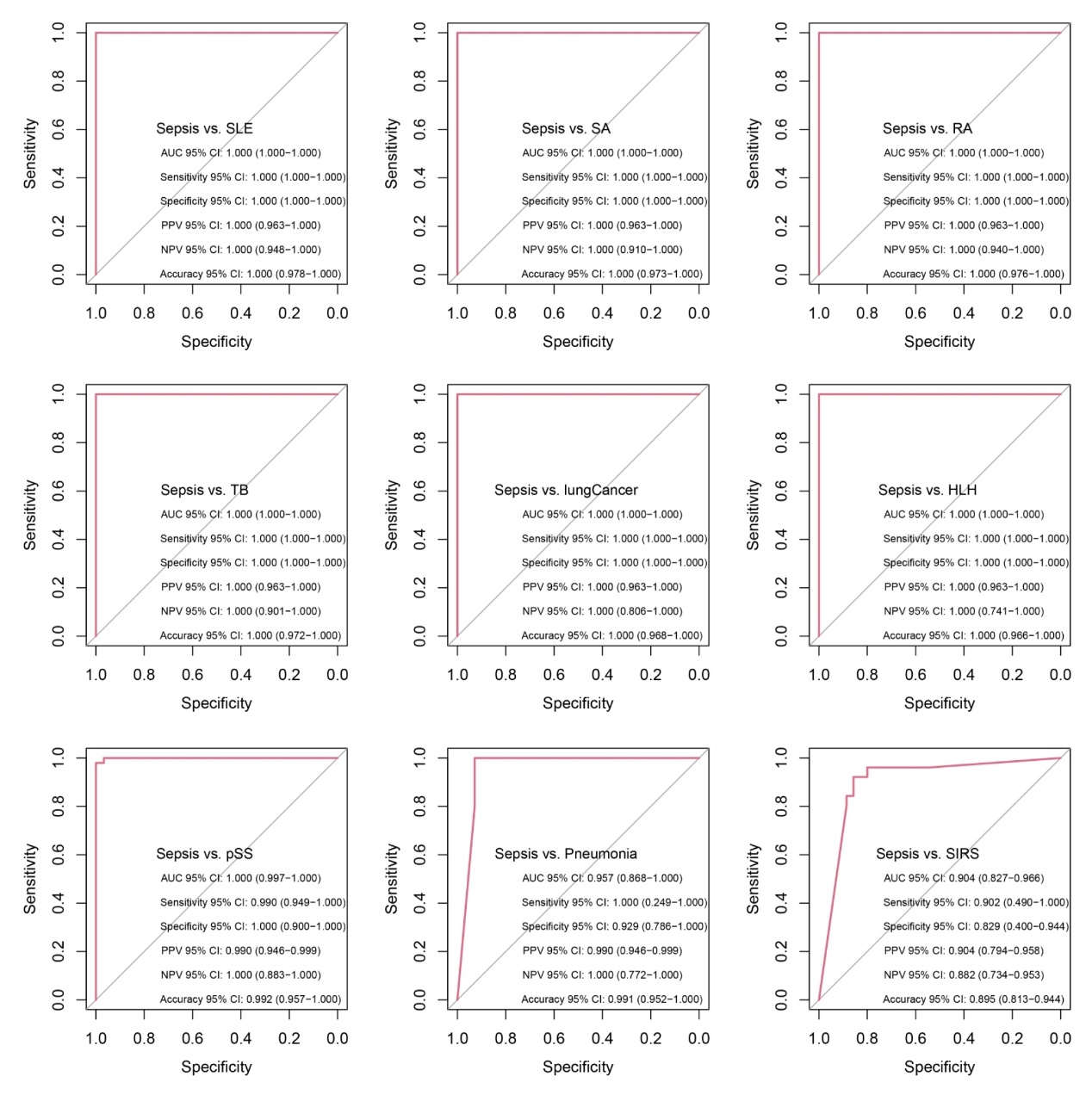


Supplementary Figure 3. Diagnostic performance of the 13-gene signature in distinguishing sepsis from OD. Receiver operator characteristic curves (ROCs) evaluating the diagnostic accuracy of the model in differentiating sepsis from various conditions, including Systemic Lupus Erythematosus (SLE), Systemic Inflammatory Response Syndrome (SIRS), Sarcoidosis (SA), Rheumatoid Arthritis (RA), Sjogren’s Syndrome (pSS), pneumonia, lung cancer, Hemophagocytic Lymphohistiocytosis (HLH), and tuberculosis (TB).


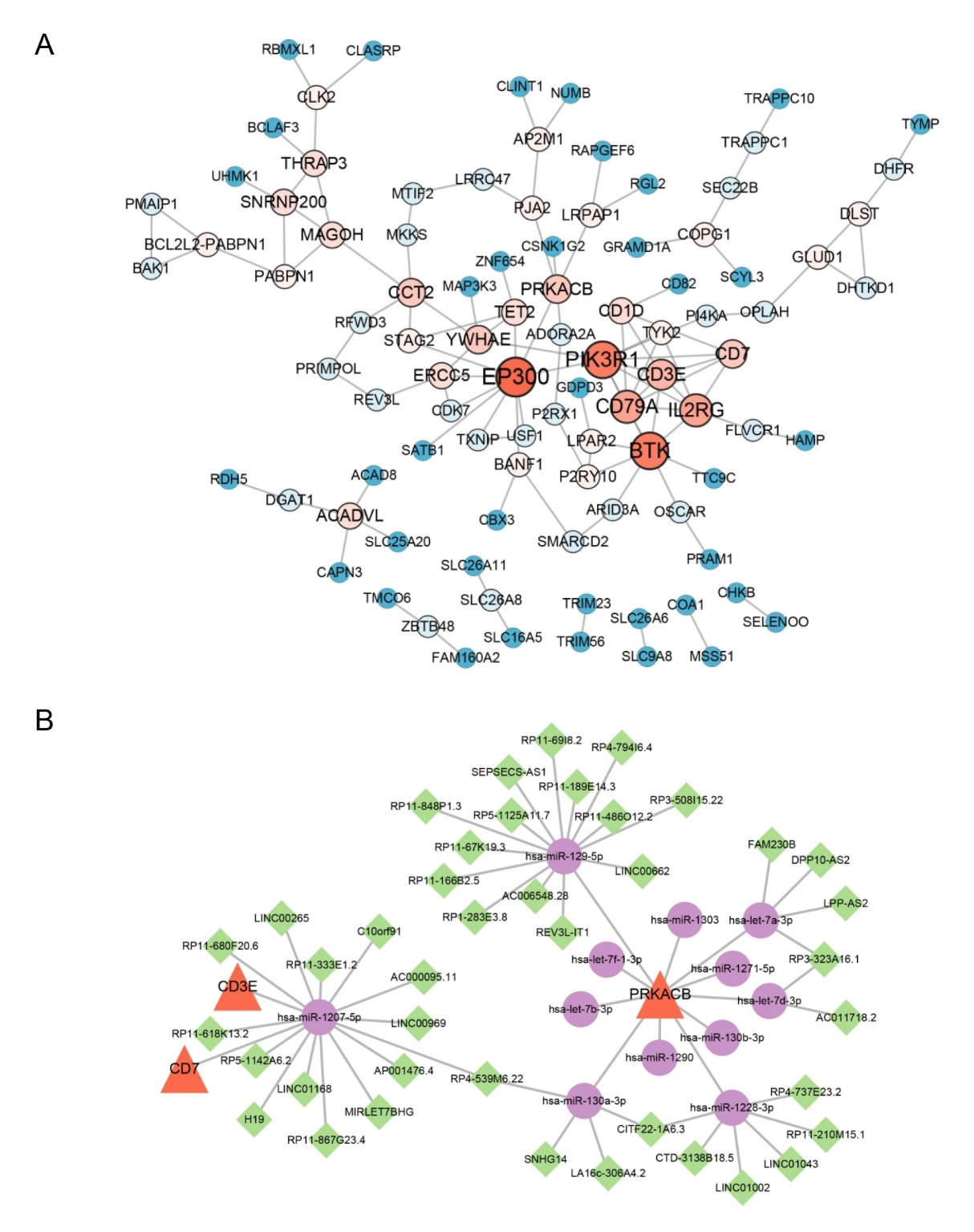


Supplementary Figure 4. Protein-Protein Interaction Analysis and construction of a ceRNA network. (A) DEGs between sepsis and SIRS, as well as SIRS and HC were subjected to PPI analysis using the STRING platform, with a medium confidence score threshold of greater than 0.8. (B) Construction of a ceRNA network centered around hub genes based on SpongScan and miRDB platforms. Red triangles represent hub genes, blue circles indicate unapproved drugs, green circles indicate approved drugs, purple circles represent miRNAs, and green diamonds indicate lncRNAs. PPI, protein-protein interaction.


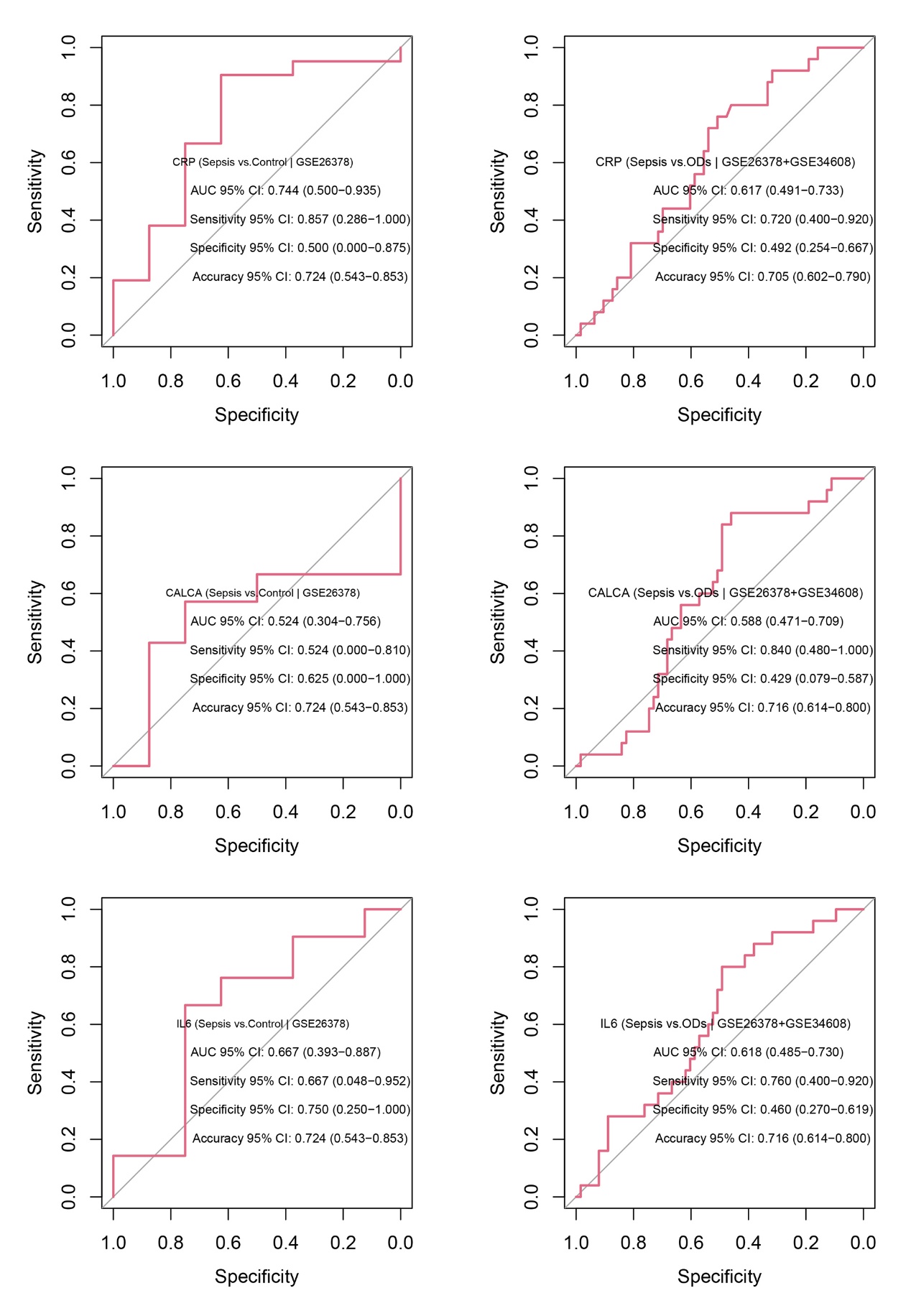


Supplementary Figure 5. Diagnostic performance of conventional sepsis biomarkers. Receiver operating characteristic (ROC) analyses of classical biomarkers C-reactive protein (CRP), procalcitonin (PCT, encoded by *CALCA*), and interleukin-6 (IL-6) in independent validation datasets.
