# Supplementary material for: Multi-omics machine learning identifies diagnostic gene signatures and functionally supports PRKACB involvement in macrophage inflammatory responses in sepsis
Source: Front Immunol. 2026 Jan 15;16:1611348. doi: 10.3389/fimmu.2025.1611348 (PMC12852012; doi:10.3389/fimmu.2025.1611348)
Supplement: Supplementary file 3 [file Table3.docx]

**Methods and Materials**

**Construction of Diagnostic Signature**

To develop diagnostic models for sepsis, the Support Vector Machine-Recursive Feature Elimination (SVM-RFE) algorithm was used to select features from variables identified in differential analysis, weighted gene co-expression network analysis (WGCNA), and univariate logistic regression. SVM-RFE was implemented using a linear-kernel support vector machine, which is appropriate for high-dimensional transcriptomic data and yields a stable and interpretable feature ranking. The cost parameter was set to 1, and features were ranked by the absolute value of the linear support vector machine weight. At each elimination step, the bottom 10% of ranked features were removed, and the procedure was repeated to generate candidate feature subsets of varying sizes.

The combined dataset was randomly split into a training cohort (70%) and a test cohort (30%) with stratification by class to preserve group proportions. All feature selection procedures were performed using the training cohort only, and the test cohort was held out for internal evaluation. Before modeling, gene expression values were standardized in the training cohort using z-score transformation, and the same centering and scaling parameters were applied to the test cohort and independent validation cohorts. To determine the optimal number of features retained by SVM-RFE, stratified 10-fold cross-validation was performed within the training cohort. For each candidate feature subset size generated along the SVM-RFE elimination path, a logistic regression classifier was fitted within each training fold and evaluated in the corresponding held-out fold. Classification performance was quantified by the mean Area Under the Curve (AUC) across folds, and the feature subset that maximized the mean AUC was selected as the final feature set.

Permutation testing was then conducted to assess whether the observed cross-validated discrimination could arise by chance. Class labels were randomly permuted 1,000 times within the training cohort, and for each permutation the same stratified 10-fold cross-validation procedure described above was repeated to obtain a null distribution of AUC values. The empirical P value was defined as the proportion of permuted AUC values greater than or equal to the observed cross-validated AUC.

Using the selected feature sets, a logistic regression model was subsequently fitted once in the full training cohort to distinguish sepsis from healthy controls (HC). For validation, GSE26378 and GSE13904 were used as independent cohorts. A second logistic regression model was fitted to differentiate sepsis from other diseases (ODs), with GSE26378, GSE72326, and GSE13904 used as validation cohorts. For both models, coefficients estimated from the discovery training cohort were fixed and directly applied to the test cohort and all independent cohorts without retraining. For each sample, the risk score was calculated as the linear predictor from the fitted logistic regression model, combining the weighted contributions of selected features using the regression coefficients. The risk score was used to generate the Receiver Operating Characteristic (ROC) curve and to calculate AUC for performance evaluation. Sensitivity and specificity were calculated using a cutoff determined in the training cohort by Youden’s index and then applied consistently to the test and validation cohorts. The final risk score formulas were:

**28-gene signature, sepsis versus healthy controls (HC)**

Risk score = (-56.240)+1.546×(IRAK3 expression)+6.743×(METTL7B expression)+0.684×(PCOLCE2 expression)+(-4.190)×(TMSB4X expression)+(-0.411)×(BMX expression)+3.013×(GRB10 expression)+(-0.130)×(PRKACB expression)+2.472×(FAM20A expression)+3.580×(KIF1B expression)+0.455×(KCNE1 expression)+(-6.673)×(CKAP4 expression)+(-0.566)×(MS4A4A expression)+0.521×(ARG1 expression)+1.745×(GPR84 expression)+1.851×(PFKFB2 expression)+0.548×(SERPINB1 expression) +3.698×(HK3 expression)+5.093×(FLOT1 expression)+2.267×(MKNK1 expression)+4.561×(UGCG expression)+(-1.157)×(GYG1 expression)+(-0.470)×(TLR5 expression)+(-1.143)×(GPR97 expression)+(-4.327)×(HGF expression)+0.208×(FCAR expression)+0.947×(SORT1 expression)+(-0.919)×(GNS expression)+(-1.191)×(SMPDL3A expression)

**13-gene signature, sepsis versus other diseases (ODs)**

Risk score = (-197.657)+1.302×(METTL7B expression)+1.567×(OLAH expression)+8.076×(PRTN3 expression)+1.888×(MMP8 expression)+4.978×(KCNE1 expression)+(-7.540)×(DACH1 expression)+15.517×(MT1M expression)+1.487×(FAM20A expression)+0.204×(ZNF404 expression)+9.440×(FAM132B expression)+2.751×(FGG expression)+1.628×(PRKACB expression)+8.832×(SUCNR1 expression)

Batch effects among GEO datasets were corrected by ComBat before merging using the sva package. The qRT-PCR validation used fixed model coefficients derived from discovery data without retraining, ensuring strict external validation.

**Single-cell RNA Sequencing Analysis**

Single-cell RNA-seq data (GSE175453) were processed using Cell Ranger (v6.1.2, 10x Genomics) with alignment to the human GRCh38 reference. For each sample, FASTQ files were quantified using cellranger count to generate filtered feature-barcode matrices, which were imported into Seurat (v5). Quality control was performed at the sample level prior to integration. Cells were retained if they contained 400–4,000 detected genes, >1,000 unique molecular identifiers (UMIs), and <20% mitochondrial reads. Genes expressed in fewer than 3 cells were excluded. Doublets were identified and removed using DoubletFinder (v2.0.3). The expected doublet number was set to 8% of recovered cells per sample, and the optimal pK parameter was selected using the standard parameter sweep approach.

Normalization and variance stabilization were performed using SCTransform with 3,000 variable features, regressing out mitochondrial content and library size metrics. Principal component analysis was conducted on the SCTransform-corrected expression matrix, and batch effects across samples were corrected using Harmony (v0.1.1) on the PCA embeddings. A shared nearest-neighbor graph was constructed using the integrated low-dimensional space, and clustering was performed using the Louvain algorithm at resolution 0.1. UMAP was used for visualization. Major immune and stromal lineages were annotated based on canonical marker genes, and myeloid cells were further subset and re-clustered to define monocytes, dendritic cells, macrophages, mast cells, neutrophils, and platelets.

Differential expression testing was performed using MAST (v1.24.0) with a minimum absolute log2 fold-change threshold of 0.25 and false discovery rate (FDR) control by the Benjamini–Hochberg method, using FDR-adjusted P < 0.05 as significance. PRKACB expression was examined across annotated cell types and within myeloid subpopulations. For PRKACB-associated transcriptional programs, cells within each condition were stratified into PRKACB-high and PRKACB-low groups using the median PRKACB expression as the cutoff, followed by differential expression and pathway enrichment analyses.
